# Toward precision oncology: deciphering the circRNA-EMT axis in cancer and its therapeutic implications

**DOI:** 10.1080/15476286.2026.2639611

**Published:** 2026-04-08

**Authors:** Zekai Lv, Guang Xie, Wenming Xu

**Affiliations:** a Joint Lab of Reproductive Medicine of SCU-CUHK, Lab of Reproductive Genetics and Epigenetics, Department of Obstetrics/Gynecology, Key Laboratory of Birth Defects and Related Disease of Women and Children of MOE, West China Second University Hospital, Sichuan University, Chengdu, China; b Department of Critical Care Medicine, Frontiers Science Center for Disease-Related Molecular Network, State Key Laboratory of Biotherapy and Cancer Center, West China Hospital, Sichuan University, Chengdu, China

**Keywords:** Circular RNAs, epithelial-mesenchymal transition, signalling pathways, tumour metastasis, biomarkers

## Abstract

The epithelial-mesenchymal transition is a pivotal driver of cancer metastasis, the leading cause of mortality in solid tumours. Circular RNAs, a unique class of endogenous RNAs characterized by covalently closed loop structures and high stability, have emerged as key regulators in this process. Accumulating evidence reveals widespread dysregulation of circRNAs during EMT, where they function as critical modulators – either promoting or inhibiting the metastatic cascade. This review systematically elucidates the mechanisms by which circRNAs govern EMT, focusing on their interactions with classical signalling pathways (TGF-β, Wnt/β-catenin, and PI3K/AKT) and core EMT-transcription factors. Furthermore, we evaluate the dual promise of circRNAs as stable, disease-specific biomarkers for liquid biopsy and as novel therapeutic targets. Deciphering the complex circRNA-EMT regulatory network not only deepens our understanding of metastasis but also provides a rational framework for developing precision oncology strategies to intercept metastatic disease.

## 1. Introduction

This review aims to systematically summarize the current understanding of how Circular RNAs (circRNAs) regulate epithelial-mesenchymal transition (EMT) pathways in cancer and explore their translational potential.

Tumour metastasis is a multi-step dynamic process in which malignant cells detach from the primary tumour, achieve local invasion by degrading the extracellular matrix through proteasomes and other mechanisms [1], enter the circulation and survive at distant sites, and ultimately colonize and grow in distant organs by acquiring induced stem cell-like properties, forming deadly secondary tumours [2]. Cancer metastasis remains the primary cause of cancer-related mortality, accounting for approximately 90% of solid tumour deaths [3]. A critical driver of this lethal process is the EMT, a reversible developmental programme that enables stationary epithelial cells to acquire migratory and invasive mesenchymal properties [4].

EMT is not merely a morphological change but profoundly impacts cancer cell behaviour [5,6]. The cell stemness and enhanced aggressiveness conferred by EMT enable cancer cells to proliferate more aggressively, survive under varied stress conditions, and evade external pressures [7]. Clinical evidence demonstrates that patients whose tumours exhibit activated EMT pathways have a considerably higher mortality rate, accounting for approximately 90% of all cancer deaths [8], underscoring the critical importance of investigating EMT in relation to poor prognosis and cancer progression. Consequently, a detailed dissection of the molecular mechanisms that govern EMT is of paramount importance for deciphering the metastatic cascade and for informing clinical prognostication in cancer therapy.

Under the current pathological classification system, EMT can be divided into three distinct types [9]. They play different roles in embryonic development, tissue repair, and cancer [10]. The EMT phenomenon is a complex biological process involving intricate networks of inducers, core regulators, and effectors that interact in a temporally coordinated manner [11]. EMT inducers mainly include transforming growth factor-β (TGF-β), Wnt/β-catenin, PI3K/AKT, and other signalling pathways. These inducers activate the expression of core EMT regulators, which in turn execute the regulatory programme [12]. The core regulators of EMT include three major families of EMT-activated transcription factors (EMT-TFs): the Snail family [13], the Zeb family [14], and the Twist family [15]. Other EMT-TFs mainly include c-Myc, FOXC2, and HIF-1α. These EMT-TFs regulate the expression of effector molecules through epigenetic mechanisms [14]. After experiencing EMT, cancer cells lose proper target recognition and activate self-sufficient growth signals to achieve metastasis while avoiding apoptosis [16].

Meanwhile, the non-coding genome, particularly circRNAs, has emerged as a key player in post-transcriptional regulation [17]. Unlike linear RNAs, circRNAs form a covalently closed loop structure, conferring high stability and making them ideal regulatory molecules [18]. Current research techniques indicated that circRNAs play widespread and pivotal roles in organisms [19] and exhibit specificity in various tissues and diseases [20,21]. Owing to their characteristic properties of high stability and specificity, circRNAs are increasingly recognized as valuable biomarker targets for assessing disease progression [22].

Previously, EMT research focused predominantly on protein-coding genes and their transcriptional regulators [23]. However, with the development of high-throughput detection technology, existing researches reveal dynamic alterations in circRNA expression during EMT [24], positioning them as novel regulators within the established EMT signalling networks.

Numerous studies have indicated that circRNAs are pervasively dysregulated across a spectrum of human diseases, with their aberrant expression patterns being particularly prominent in malignant tumours [25]. This widespread dysregulation suggests that circRNAs may play a critical role in disease pathogenesis and progression. Notably, the EMT – a fundamental biological process driving cancer invasion and metastasis – has been found to be functionally linked to circRNA expression [26]. High-throughput RNA sequencing analyses investigating the role of circRNAs in EMT have revealed that the expression levels of hundreds of circRNAs are significantly altered during EMT, with the vast majority being specifically upregulated [27]. This finding strongly implies that EMT may represent a crucial pathway through which circRNAs govern cancer development.

Existing research demonstrates that circRNAs indirectly regulate EMT through complex molecular interactions [28]. It is well-established that multiple oncogenic signalling pathwayscan undergo aberrant activation or functional inactivation due to dysregulated miRNA activity, thereby acting as triggers for EMT [29]. Given that circRNAs are widely recognized as competing endogenous RNAs (ceRNAs) that function as efficient ‘molecular sponges’ for miRNAs [30], they indirectly modulate the excessive promotion or suppression of EMT-related genes targeted by these miRNAs through this mechanism [31]. Consequently, circRNAs exert a profound influence on cancer cell migration, invasion, and distant metastatic capability.

CircRNAs, characterized by their notable stability and resistance to degradation, have recently emerged as a prominent area of investigation in cancer research [32]. Current studies in the field of circRNAs in cancer therapeutics are primarily centred around three key directions: (1) serving as therapeutic targets; (2) modulating the tumour microenvironment and immune responses; and (3) functioning as diagnostic and prognostic biomarkers [33]. With the deepening understanding of circRNA biology, research on circRNAs in cancer treatment has progressively advanced from fundamental mechanistic exploration to clinical translation, thereby paving novel pathways for the precise diagnosis and targeted therapy of cancers [34].

**Figure 1.**
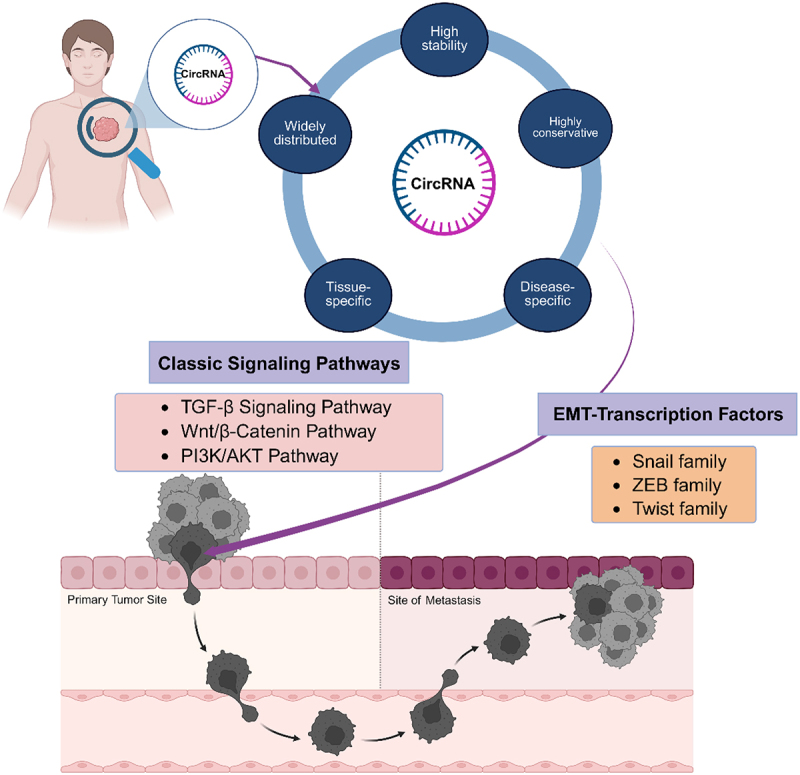
CircRNAs: emerging role in EMT regulation and precision oncology. Circular RNA is characterized by high stability and strong specificity. CircRNAs effectively modulate key signaling pathways and EMT transcription factors, critically governing the initiation, maintenance, and metastasis of epithelial-mesenchymal transition in cancer. These properties make circRnas promising biomarkers and therapeutic targets for precision oncology.

This review systematically summarizes the molecular mechanisms by which circRNAs regulate EMT in cancer, with a focus on elucidating their modulatory roles via key signalling pathways – including TGF-β, Wnt/β-catenin, and PI3K/AKT – as well as EMTrelated transcription factors. It further evaluates the potential of circRNAs as highly stable, tissuespecific liquid biopsy biomarkers for cancer diagnosis and prognosis, and examines their feasibility as therapeutic targets along with current circRNAbased interventional strategies. Finally, the review highlights existing challenges in research models, detection methods, and clinical translation, and proposes that technological innovation, standardization, and intelligent design are essential to advance circRNA research towards precision oncology, thereby consolidating the foundation for developing novel diagnostic and therapeutic approaches. (Figure 1)

## 2. Overview of EMT

### 2.1. Molecular characteristics and types of EMT

EMT is a reversible embryonic cell process in which epithelial cells lose their unique characteristics such as apical polarity, epithelial markers, and intercellular junctions. During this process, cells undergo cytoskeletal structural reorganization and dedifferentiation, returning to a mesenchymal phenotype with enhanced migration and invasion capabilities [35]. This transformation is characterized by coordinated molecular changes: epithelial markers including E-cadherin, claudin, occludin, and cytokeratin are downregulated, while mesenchymal markers such as fibronectin, vimentin, integrin-β6, and N-cadherin are upregulated.

Under the existing pathological classification system, EMT can be divided into three types based on biological context and functional outcomes [9]. Type I EMT plays important physiological roles in embryonic development and organogenesis, such as gastrulation and outward migration of cells diverging from neural ridges, contributing to proper tissue patterning during development. Type II EMT plays important roles in wound healing and tissue repair, such as inducing cell migration and growth. However, when dysregulated, Type II EMT can lead to pathological organ fibrosis in tissues such as liver, kidney, and lung. Type III EMT affects the occurrence and progression of various diseases, most notably cancer. Through Type III EMT, epithelial cancer cells transform into mesenchymal cancer cells and metastasize to distant organs of the human body, representing a critical step in cancer progression and a major clinical challenge.

### 2.2. The regulatory cascade of EMT

EMT is a complex process coordinately driven by inducers, core regulators, and effector molecules. EMT inducers, including TGF β, Wnt/β catenin, and PI3K/AKT signalling pathways, initiate the programme and converge on core EMT TFs [36]. These master regulators are categorized into three major families: the Snail family [9], the Zeb family [14], and the Twist family. These EMT TFs regulate the expression of downstream effector molecules through multiple mechanisms, such as direct transcriptional control and epigenetic modifications. The functional outcomes of EMT are executed by effector molecules, characterized by downregulation of epithelial markers and upregulation of mesenchymal markers [37]. These alterations lead to fundamental changes in cell morphology, adhesion, and motility. In cancer, EMT enables loss of targeting accuracy, activation of autonomous growth signalling, metastasis, and evasion of apoptosis [16]. The resulting enhanced survival and migratory capacity facilitate dissemination from primary tumours and the formation of lethal distant metastases.

Beyond direct regulation, circRNAs also indirectly influence EMT and metastasis by modulating interconnected cell fate-determining processes such as autophagy, necroptosis, and pyroptosis [35,38]. Autophagy and EMT engage in extensive mutual regulation, where autophagy-related proteins (e.g. ATG5 [39], ATG12 [40]) promote EMT, and EMT transcription factors like ZEB1 can enhance autophagic activity [41], forming a metastasis-driving feedback loop. CircRNAs can alter autophagic flux by sponging miRNAs or binding RBPs, thereby regulating the EMT phenotype [42]. Similarly, circRNAs can regulate key executors of necroptosis or pyroptosis [43], which are programmed pro-inflammatory cell death forms that remodel the tumour immune microenvironment through cytokine release [44–46]. This remodelling can indirectly induce EMT in neighbouring cells via pathways such as NF-κB [47], suggesting a circRNA-mediated axis of ‘regulating cell death modalities —— influencing the tumour microenvironment —— indirectly modulating EMT’.

### 2.3. The association between EMT and cancer stem cells

The EMT is not only a critical step for cancer cells to acquire migratory and invasive capabilities but also a core biological process that remodels cell fate and confers stem-like properties [37]. Cancer stem cells (CSCs), also known as tumour-initiating cells, represent a small subpopulation within tumours that possess self-renewal, multipotent differentiation potential, and high tumorigenic capacity. They are considered the cellular root of tumour initiation, metastasis, recurrence, and therapy resistance [48].

The EMT programme and CSC characteristics exhibit profound convergence and synergy at the molecular level. Studies have found that cells undergoing EMT frequently upregulate a series of core pluripotency transcription factors and cell surface markers, thereby acquiring a typical CSC phenotype [49]. This process is primarily driven by core EMT-transcription factors. For instance, SNAIL and ZEB family proteins can not only suppress epithelial markers such as E-cadherin but also directly transcriptionally activate stemness-related gene networks. TWIST, on the other hand, can maintain a dedifferentiated state and cellular plasticity by regulating epigenetic modifiers like Bmi-1 [50]. This sharing of regulatory networks implies that EMT, while inducing changes in cell morphology and motility, fundamentally reprograms the biological identity of the cell, steering it towards a more aggressive, plastic, and therapy-resistant CSC state [51].

## 3. Structural characteristics and biological functions of circRNA

### 3.1. Genomic landscape and circRNA discovery

Although around 93% of DNA sequences in the human genome can be transcribed into RNA, less than 2% of nucleic acid sequences are used to encode proteins [52]. The remaining transcripts are non-coding RNAs (ncRNAs) that do not encode proteins. NcRNAs lack open reading frames and cannot encode proteins through conventional mechanisms, yet they function as crucial regulators of various biological processes, including development, proliferation, transcription, and post-transcriptional modification. Among the diverse types of ncRNAs, microRNA (miRNA), long non-coding RNA (lncRNA), and circular RNA (circRNA) have been widely recognized for their roles in innate immune regulation and disease pathogenesis.

Circular RNA was discovered approximately 40 years ago and is widely present in fungi, protozoa, plants, and humans [53]. Initially, circRNAs were considered to be splicing error products with minimal biological significance. However, with the application of high-throughput RNA-seq techniques, circRNAs have been demonstrated to play widespread and pivotal roles in organisms and exhibit remarkable specificity in various tissues and disease states [20,21]. This tissue and disease specificity suggests that circRNAs are precisely regulated molecules with important biological functions rather than merely transcriptional byproducts.

### 3.2. Unique structural features of circRNAS

CircRNAs represent a special class of ncRNAs that differ fundamentally from other linear RNAs in their molecular architecture. Unlike linear mRNAs, circRNAs lack the 5’cap structure and 3’ poly(A) tail, and instead exist in a covalently closed loop configuration [54]. This unique circular structure has profound implications for circRNA stability and function. Since the circular structure lacks free ends, circRNAs are not degraded by RNA exonucleases that typically process linear RNAs from their terminal ends. Consequently, circRNAs exhibit stable expression patterns and accumulate to relatively high steady-state levels in cells, making them particularly suitable for long-term regulatory functions [55].

CircRNAs arise via back-splicing of precursor mRNAs, forming covalently closed loops. In this process, RNA polymerase II transcribes precursor mRNA, but instead of conventional forward splicing, the 3’end splice site is joined to an upstream 5’ end splice site via back-splicing to form a closed loop structure [56]. This back-splicing mechanism can be facilitated by complementary sequences in flanking introns that bring distant splice sites into proximity, or by RNA-binding proteins that bridge different regions of the pre-mRNA molecule. Based on their genomic origin and the composition of the circular transcript, circRNAs can be systematically classified into three major categories [57]: exonic circRNAs (EcRNAs), which consist solely of exonic sequences; intronic circRNAs (CiRNAs), which are composed entirely of intronic sequences; and exon-intron circRNAs (ElcRNAs), which contain both exonic and intronic sequences within the circular molecule.

### 3.3. Molecular mechanisms of circRNA function

The biological functions of circRNAs are mainly categorized into four distinct molecular mechanisms [58], each contributing to gene regulation in unique ways:(1) MicroRNA sponge function: This represents the most extensively studied mechanism of circRNA action. CircRNAs possess multiple binding sites for miRNAs, functioning as competing endogenous RNAs (competing endogenous RNA, ceRNAs). By sequestering miRNAs through complementary base pairing, circRNAs prevent these miRNAs from binding to their target mRNAs, thereby indirectly regulating gene expression by inhibiting miRNA-mRNA interactions. The circular structure and stability of circRNAs make them particularly effective as miRNA sponges, as they can accumulate to high concentrations and persistently sequester miRNAs over extended periods. (2) Regulatory protein binding: CircRNAs can interact with RNA-binding proteins (RBPs) that are involved in mRNA regulation, thereby modulating various aspects of RNA metabolism. Through these interactions, circRNAs can alter the stability, localization, or splicing patterns of target mRNAs. Some circRNAs function as scaffolds that bring multiple proteins together, while others sequester RBPs away from their normal target RNAs, thereby modulating cellular processes through protein sequestration. (3) Protein coding capacity: Although circRNAs are classified as non-coding RNAs and generally do not participate in translation through conventional cap-dependent mechanisms, a small proportion of circRNAs can be translated into functional peptides. This translation can occur through internal ribosome entry sites (IRES) or be driven by N^6^-methyladenosine (m^6^A) modifications. Although this represents a minor fraction of circRNA functions, the peptides produced can regulate important cellular processes including transcription and signalling. (4) Gene transcription regulation: CircRNAs, particularly those localized to the nucleus, can directly or indirectly interact with RNA polymerase II and other transcriptional machinery components to regulate gene transcription. This function allows circRNAs to influence gene expression at the transcriptional level, complementing their more prevalent post-transcriptional regulatory roles.

### 3.4. CircRNAs in cancer and EMT

Studies have demonstrated that circRNAs are dysregulated in various diseases, with cancer being among the most extensively studied [25]. Comprehensive expression profiling has revealed widespread circRNA dysregulation across multiple cancer types, with altered circRNA expression often correlating with disease stage, metastatic potential, and patient prognosis. EMT, as an essential molecular process in cancer metastasis, has been shown to have important relationships with circRNA expression patterns.

**Figure 2.**
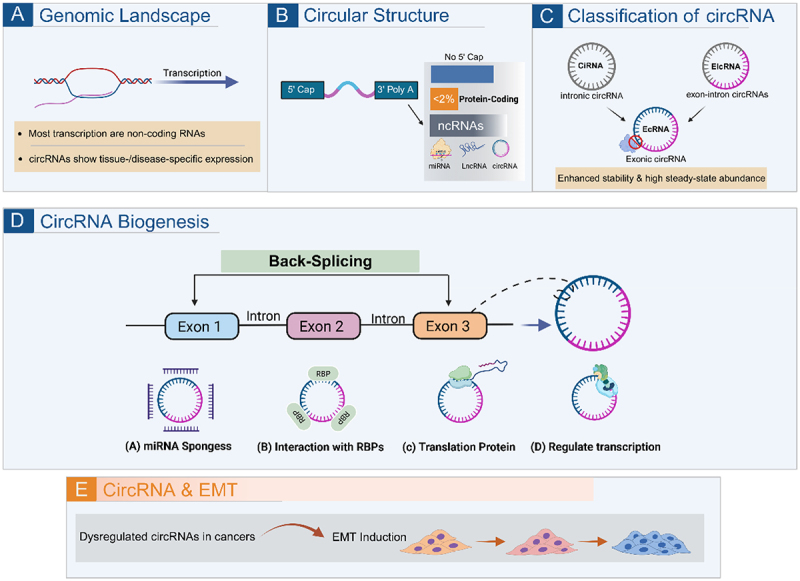
Biogenesis and functional mechanisms of circular RNA. (A) Genomic landscape. (B) Circular structure. (C) Classification of circRNA. (D) CircRNA biogenesis. (E) CircRNA can participate in the EMT process of cancer cells.

A pivotal study examining the role of circRNAs in EMT conducted high-throughput RNA sequencing analysis, demonstrating that the expression of hundreds of circRNAs is dynamically regulated during EMT induction [27]. Notably, most of these differentially expressed circRNAs were upregulated during the EMT process, suggesting that circRNAs may predominantly function as pro-EMT regulators, although important EMT-suppressive circRNAs have also been identified. This observation indicates that EMT may be an essential pathway through which circRNAs control cancer development and progression. The functional significance of these expression changes is supported by mechanistic studies demonstrating that circRNAs can modulate EMT through regulation of key signalling pathways and transcription factors, ultimately affecting cancer cell migration, invasion, and metastatic capacity. (Figure 2)

## 4. Regulation of EMT by circRNA

CircRNAs regulate EMT through various molecular mechanisms, which can be systematically categorized based on the signalling pathways and molecular targets they modulate. The following sections examine how circRNAs interface with major EMT-regulatory pathways.

### 4.1. TGF-β signaling pathway

Studies have demonstrated that dysregulation of the TGF-β pathway is associated with the occurrence of EMT in various cancers, including cervical cancer and lymphoma [59,60]. The TGF-β signalling pathway represents one of the most potent inducers of EMT across diverse cellular contexts. Within this pathway, Smad3, a member of the Smad family, has been identified as one of the most pivotal terminal downstream factors in TGF-β signalling transduction [61].

CircRNA-mediated regulation of Smad3 stability serves as a pivotal mechanism in cancer progression, as exemplified by the axis of hsa_circ_0088036/miR-1343-3p/Bcl-3 in NSCLC, wherein this circRNA sponges miR-1343-3p to derepress the proto-oncogene Bcl-3, whose protein product directly interacts with the MH2 domain of Smad3. This interaction competitively blocks the binding of E3 ubiquitin ligases or adapters to this critical domain, thereby inhibiting Smad3 ubiquitination and proteasomal degradation, ultimately enhancing its stability and amplifying the TGF-β/Smad3/EMT signalling cascade [62]. This conserved paradigm is recapitulated in other malignancies with subtype-specific nuances, such as in the diffuse subtype of gastric cancer where circNRIP1 acts as an oncogene by sponging miR-149-5p to upregulate FOXM1, which in turn facilitates Smad3 phosphorylation and nuclear translocation, thereby potentiating the TGF-β-induced EMT programme [63]. This subtype-specificity, further evidenced by circ-HER2 stabilizing Smad3 and promoting EMT predominantly in hormone receptor-positive breast cancer, underscores how cellular context dictates circRNA network functions [64]. Transitioning to clinical translation, while direct therapeutic targeting of such oncogenic circRNAs remains largely preclinical, strategies like antisose oligonucleotides show promise in animal models. The clinical application is currently more advanced in diagnostics, where circRNAs involved in this axis, such as hsa_circ_0000567 in colorectal cancer, have been validated as promising plasma biomarkers for early detection and metastasis prediction in clinical cohorts [65].

CircRNA-mediated regulation of transcriptional intermediary factor 1γ (TIF1γ) exemplifies a sophisticated network-based mechanism for suppressing EMT. Transcriptional intermediary factor 1γ (TIF1γ) is a protein that inhibits TGF-β/Smad signalling by promoting Smad4 ubiquitination and competing with Smad4 for binding to the Smad2/3 complex [66]. CircPTK2 promotes TIF1γ expression by sponging miR-429 and miR-200b-3p, which normally suppress TIF1γ by targeting its 3’-UTR. By upregulating TIF1γ, circPTK2 inhibits TGF-β/Smad signalling transduction, thereby suppressing EMT [67]. Beyond regulating the TGF-β/Smad signalling axis, TIF1γ also exhibits additional EMT-suppressive functions. TIF1γ competes with TATA box-binding protein (TBP) and TBP-associated factor 15 (TAF15), impeding TAF15/TBP-mediated interleukin-6 (IL-6) transactivation. Mechanistically, TIF1γ drives the nuclear export of TAF15 through multiple monoubiquitination modifications of TAF15 [68]. Functionally, TAF15 accelerates epithelial-mesenchymal transition and metastasis of lung adenocarcinoma cells, acting in an opposite manner to TIF1γ. These findings indicate that the TAF15/TBP complex is essential for IL-6 activation-induced EMT and invasion, while TIF1γ inhibits these processes by antagonizing this complex. This positions circPTK2, through TIF1γ upregulation, as a critical node suppressing both TGF-β/Smad-dependent and IL-6-dependent EMT signalling branches.

In the context of positive feedback loops in circRNA-TGF-β regulation, the downstream regulation of circRNA function is primarily governed by competitive endogenous RNA mechanisms [69], which are relatively well-defined. However, the mechanisms underlying the upstream regulation of circRNA biogenesis remain incompletely understood [70]. It has been reported that alternative splicing factors and RNA-binding proteins (RBPs) may be involved in the upstream regulation of circRNA production [71].

**Figure 3.**
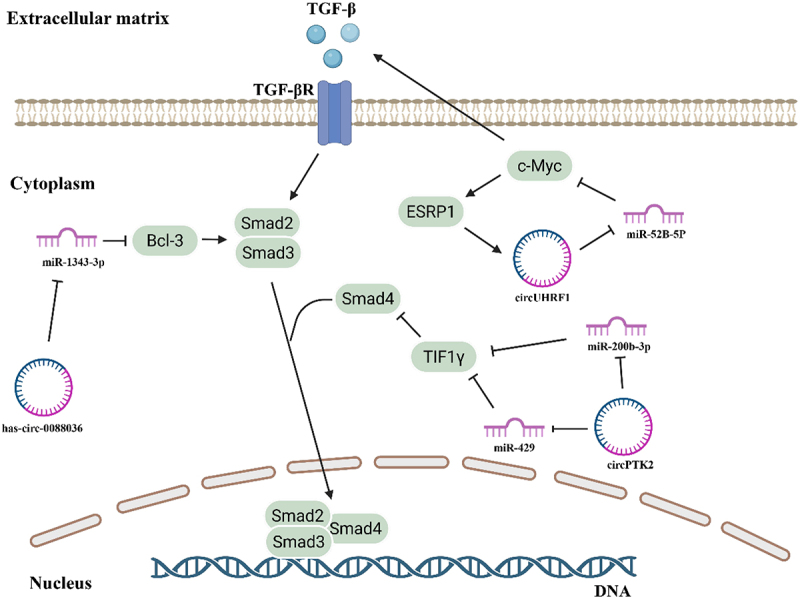
CircRNA-mediated regulation of EMT through the TGF-β signaling pathway. CircRNAs regulate EMT through multiple mechanisms in the TGF-β pathway. Hsa_circ_0088036 promotes EMT by sponging miR-1343-3p, leading to increased Bcl-3 expression, which stabilizes Smad3 and activates TGF-β/Smad3 signaling. CircPTK2 inhibits EMT by sponging miR-429 and miR-200b-3p, resulting in elevated TIF1γ expression, which suppresses TGF-β/Smad signaling by promoting Smad4 degradation. CircUHRF1 forms a positive feedback loop by sponging miR-526b-5p, which upregulates c-Myc. c-Myc then promotes both TGF-β1 expression (activating EMT) and ESRP1 expression (a splicing factor that enhances circUHRF1 biogenesis), thereby creating a self-reinforcing regulatory circuit.

In oral squamous cell carcinoma (OSCC), a sophisticated self-reinforcing regulatory circuit with the circRNA at its core has been elucidated, involving circUHRF1 (hsa_circ_0002185). In this system, miR-526b-5p binds to c-Myc’s 3’-UTR to inhibit its expression, while circUHRF1 can sponge miR-526b-5p, thereby positively regulating c-Myc [72]. The transcription factor c-Myc then accelerates the transcription of two key targets: TGF-β1, which promotes EMT, and epithelial splicing regulatory protein 1 (ESRP1) [73]. Importantly, the splicing factor ESRP1 promotes UHRF1 gene cyclization and biogenesis by targeting the flanking introns of circUHRF1. This completes a closed, autoregulatory loop where circUHRF1 initiates and is itself regenerated by the pathway it activates, solidifying its central regulatory role. This positive feedback loop of circUHRF1/miR-526b-5p/c-Myc/TGF-β1/ESRP1 accelerates OSCC development and EMT through the TGF-β1 pathway. Furthermore, analogous regulatory mechanisms involving ESRP1 and TGF-β1 have been identified in breast cancer. In this context, the mechanism can be targeted by the transcription factor upstream transcription factor 1 (USF1). The circular RNA circANKS1B accelerates EMT formation by targeting miR-148a and miR-152-3p, which activate expression of the transcription factor USF1. USF1 then promotes TGF-β and ESRP1 expression, generating a similar feedback loop [74]. These examples demonstrate that circRNAs can function as pivotal orchestrators within positive feedback loops where the TGF-β pathway synergizes with upstream and downstream factors, creating self-reinforcing circuits that stabilize the mesenchymal phenotype. (Figure 3)

### 4.2. Wnt/β-catenin pathway

Carcinogenesis is frequently accelerated by inactivation of tumour suppressor genes such as adenomatous polyposis coli (APC) in the Wnt signalling pathway, or by oncogenic mutations in β-catenin itself. High expression levels of β-catenin signalling can be detected in tumour cells at the invasion front or in cells migrating to adjacent stroma. This differential pattern of Wnt signalling activity indicates distinct functional roles: promoting both the proliferation capacity and the EMT potential of cancer cells [75].

CircRNA-mediated regulation of APC2 represents a pivotal mechanism for controlling the Wnt/β-catenin signalling axis, as exemplified in colorectal cancer (CRC) by the hsa_circ_0009361/miR-582/APC2 regulatory axis [76]. Herein, hsa_circ_0009361 functions as a critical network hub by sponging the oncogenic miR-582, thereby relieving the post-transcriptional repression of APC2—a key negative regulator of the Wnt pathway. The subsequent upregulation of APC2 protein promotes the destabilization and degradation of cytoplasmic β-catenin, inhibiting its nuclear translocation and the transcriptional activation of pro-EMT and proliferative targets such as c-Myc and cyclin D1. Thus, this circRNA-directed network effectively suppresses the Wnt/β-catenin-EMT programme [77]. The clinical relevance of this axis is underscored by the consistent downregulation of hsa_circ_0009361 in CRC tissues, where its expression inversely correlates with advanced tumour stage and metastasis, highlighting its prognostic biomarker potential. Therapeutically, while direct targeting remains preclinical, restoring the function of such tumour-suppressive circRNAs or deploying antisense oligonucleotides against their cognate oncogenic miRNAs (e.g. miR-582) represents a promising network-based strategy to impede Wnt-driven EMT and CRC progression.

CircRNAs function as critical regulatory molecules in radiation-resistant cancers by modulating the Wnt/β-catenin signalling axis to influence EMT. For instance, radiation-resistant oesophageal squamous cell carcinoma (ESCC) cells demonstrate high EMT potential. Acting as a central node in a competing endogenous RNA network, circRNA_100367 can sponge miR-217, thereby de-repressing its target Wnt3. This interaction attenuates the EMT capacity of ESCC radiation-resistant cells through the miR-217/Wnt3 regulatory axis [78]. This finding not only delineates a precise molecular mechanism driven by circRNA_100367 but also suggests that this circRNA may serve as a potential therapeutic target to overcome radiation resistance in ESCC by disrupting a key signalling cascade.

CircRNA regulation by inflammatory cytokines represents a key mechanism through which the tumour microenvironment influences cancer progression, highlighting circRNAs as dynamic mediators of extracellular signals. In CRC cells, circ_0026344 can be downregulated by chemokine (C-C motif) ligand 20 (CCL20) and C-X-C motif chemokine ligand 8 (CXCL8), which are abundant in the tumour microenvironment. This downregulation attenuates the critical sponging effect of circ_0026344 on miR-183 [79]. MiR-183 is considered a typical oncogene [80], and Wnt/β-catenin signalling is believed to be a downstream transduction pathway for miR-183. Therefore, in CRC, when circ_0026344 is inhibited by CCL20 and CXCL8, the loss of this circRNA’s regulatory function promotes EMT through activation of the Wnt/β-catenin signalling pathway [81]. This example illustrates how inflammatory signals modulate circRNA expression to indirectly affect EMT, thereby positioning specific circRNAs as central sensors and integrators of oncogenic inflammatory cues.

CircRNAs play a critical regulatory role in melanoma progression by modulating key oncogenic pathways, as exemplified by circRNA_0082835 [82]. In melanoma, circRNA_0082835 is markedly overexpressed, and its knockdown has been shown to inhibit Wnt/β-catenin pathway activity, directly linking this circRNA to a core EMT-driving signalling axis [83]. Bioinformatic analysis from the ENCORI database indicates that circRNA_0082835 contains binding sites for miRNA-429, a well-characterized tumour suppressor known to block melanoma progression [84]. Therefore, it can be inferred that circRNA_0082835 likely functions as an oncogenic driver by sponging and inhibiting miR-429, thereby derepressing the Wnt/β-catenin pathway to promote EMT. This positions circRNA_0082835 as a central ceRNA network hub that actively sustains a pro-metastatic signalling circuit in melanoma.

Direct protein interactions represent a key mechanism whereby circRNAs regulate the Wnt/β-catenin pathway, highlighting their versatile role as direct molecular scaffolds and modulators of signalling components. Beyond their function as microRNA sponges, some circRNAs can directly interact with proteins in the Wnt/β-catenin pathway. For example, circ-glycogen synthase kinase-3 beta (GSK3β), derived from the GSK3β gene, directly interacts with GSK3β protein, an upstream regulatory factor of GSK3β/β-catenin signalling. This RNA-protein interaction effectively sequesters GSK3β, preventing it from executing its normal inhibitory function and thereby promoting β-catenin activity in ESCC cells [85]. Similarly, a novel 370 amino-acid β-catenin isoform generated from circβ-catenin through translation can directly interact with GSK3β. This interaction inhibits GSK3β-mediated β-catenin phosphorylation and degradation, demonstrating how protein-coding circRNAs can produce functional antagonists of core pathway enzymes [86]. These studies demonstrate that circRNAs can regulate Wnt/β-catenin signalling through multiple mechanisms: they can function as microRNA sponges to indirectly modulate pathway components, or they can serve as core direct regulators by interacting with signalling molecules through either RNA-protein interactions or by encoding regulatory peptides, thereby actively controlling cell function and EMT.

### 4.3. Phosphoinositide-3-kinase (PI3K)/protein kinase B (AKT) pathway

The PI3K/AKT pathway is a pivotal kinase cascade that controls essential cell functions, including proliferation, transcription, translation, survival, and growth. Abnormal expression of the PI3K/AKT signalling pathway has been observed in many diseases, including breast cancer, lung cancer, and thyroid cancer [87].

CircRNA-mediated regulation of casein kinase II catalytic subunit alpha (CSNK2A1) represents a direct mechanism for activating oncogenic signalling pathways, as exemplified in thyroid cancer by the interaction between circNDST1 and CSNK2A1 [88]. In this context, circNDST1 functions not merely as a sponge but as a critical protein-binding partner, directly interacting with CSNK2A1 to enhance its kinase activity. This circRNA-protein complex significantly potentiates the phosphorylation of a key downstream substrate, AKT. The hyperphosphorylation of AKT leads to the constitutive activation of the PI3K/AKT signalling axis, a major driver of cell survival, proliferation, and notably, the EMT programme in thyroid cancer cells [89,90]. This positions circNDST1 as a central upstream regulator that amplifies a pro-metastatic signalling network through direct protein modulation. The clinical relevance of this axis is supported by the co-upregulation of circNDST1 and CSNK2A1 in aggressive thyroid cancer subtypes, correlating with advanced disease stages and poorer prognosis, thus highlighting their combined potential as a prognostic biomarker signature [91]. Therapeutically, disrupting the specific circNDST1-CSNK2A1 interaction or downstream AKT activation presents a promising strategy to inhibit PI3K/AKT-driven EMT and tumour progression in thyroid cancer.

The circGRAMD1B/SOX4/MEX3A axis plays a critical role in lung adenocarcinoma (LUAD), initiating a multi-tiered oncogenic signalling cascade. In LUAD, miR-4428 binds to the 3’-UTR region of SOX4 to inhibit SOX4 expression, while circGRAMD1B serves as the upstream master regulator by sponging miR-4428, thus derepressing SRY-Box Transcription Factor 4 (SOX4) expression [92]. SOX4, a member of the group C subfamily of SOX transcription factors, has important regulatory functions in cancer metastasis and EMT development [93]. Studies have shown that elevated SOX4 is strongly correlated with mex-3 RNA binding family member A (MEX3A), as SOX4 promotes MEX3A expression by binding to its promoter. Subsequently, MEX3A, as an mRNA-binding protein, binds to LAMA2 mRNA to reduce its stability and decrease LAMA2 expression. Given that PTEN is a classical inhibitor of the PI3K/AKT pathway and LAMA2 can significantly increase PTEN gene expression, MEX3A ultimately promotes LUAD metastasis and EMT by inhibiting LAMA2 to activate the PI3K/AKT pathway. This delineates a comprehensive circRNA-orchestrated network, forming the circGRAMD1B/miR-4428/SOX4/MEX3A/LAMA2 regulatory axis in LUAD [94].

CircRNAs serve as pivotal upstream regulators of the PI3K/AKT signalling pathway across various cancer types, orchestrating diverse pro- or anti-tumorigenic networks. In OSCC, circRNAHIPK3 acts as a central oncogenic hub by sponging the tumour suppressor miR-637, thereby derepressing nuclear protein 1 (NUPR1) expression and activating the downstream NUPR1/PI3K/AKT pathway [95]. Similarly, circPIP5K1A functions as a critical metastatic driver by serving as a competing endogenous RNA for miR-515-5p, which leads to the upregulation of TCF12 and subsequent activation of the PI3K/AKT pathway to promote cancer metastasis and EMT [96]. Conversely, hsa_circRNA_100269 exemplifies a tumour-suppressive circRNA that inhibits gastric cancer progression; its overexpression induces G0/G1 cell cycle arrest and promotes apoptosis [97], suggesting its core regulatory function is likely mediated through suppressing the oncogenic PI3K/AKT axis to inhibit metastasis and EMT.

### 4.4. CircRNA regulation of EMT-transcription factors (EMT-TFs)

The transcription factors of EMT play pivotal roles in the activation and maintenance of the EMT pathway. Their primary functions are to inhibit the transcription of epithelial marker proteins, such as E-cadherin, and promote the translation of mesenchymal marker proteins, such as N-cadherin [98]. The EMT-TFs including TWIST1, TWIST2, SNAIL1, SNAIL2, ZEB1, and ZEB2 have all been extensively studied [99].

CircRNAs function as central regulators of EMT by modulating the stability of key EMT transcription factors like SNAIL through ceRNA networks. Studies have demonstrated that miRNAs can regulate EMT-TFs, and circRNAs, acting as master miRNA sponges within these networks, can indirectly govern EMT-TF activity, thus critically affecting the EMT capacity of cancer cells [100]. In melanoma, circRNA_0084043 was reported to be upregulated. The 3’-UTR of Snail shares the same binding site for the tumour-suppressive miR-153-3p as circRNA_0084043 [82]. By sequestering miR-153-3p, circRNA_0084043 effectively derepresses Snail expression, which suggests that this circRNA promotes melanoma cell growth and metastasis by orchestrating a miR-153-3p/Snail regulatory axis [101]. Similarly, in urothelial carcinoma of the bladder, circPRMT5 serves as an oncogenic hub that competitively upregulates Snail expression by sponging miR-30c, thus inhibiting E-cadherin expression and promoting EMT [102].

CircRNA regulation of ZEB transcription factors represent a critical upstream mechanism within the established double-negative feedback loops that dynamically control EMT status [103]. In clear cell renal cell carcinoma, ZEB2 was observed to be a direct target of miR-153, which inhibits the pro-EMT effect of ZEB2. Functioning as a central regulatory node within this network, circPCNXL2 blocks miR-153’s interaction with ZEB2 by sponging miR-153. The finding that miR-153 inhibitors reversed the effects of circPCNXL2 on RCC cells confirms the axis’s functionality. These findings indicate that circPCNXL2 acts as a pivotal oncogenic circRNA and a master upstream regulator that actively promotes EMT in RCC by coordinating the miR-153/ZEB2 axis, thereby positioning circRNAs as key drivers of this oncogenic switch [104].

**Figure 4.**
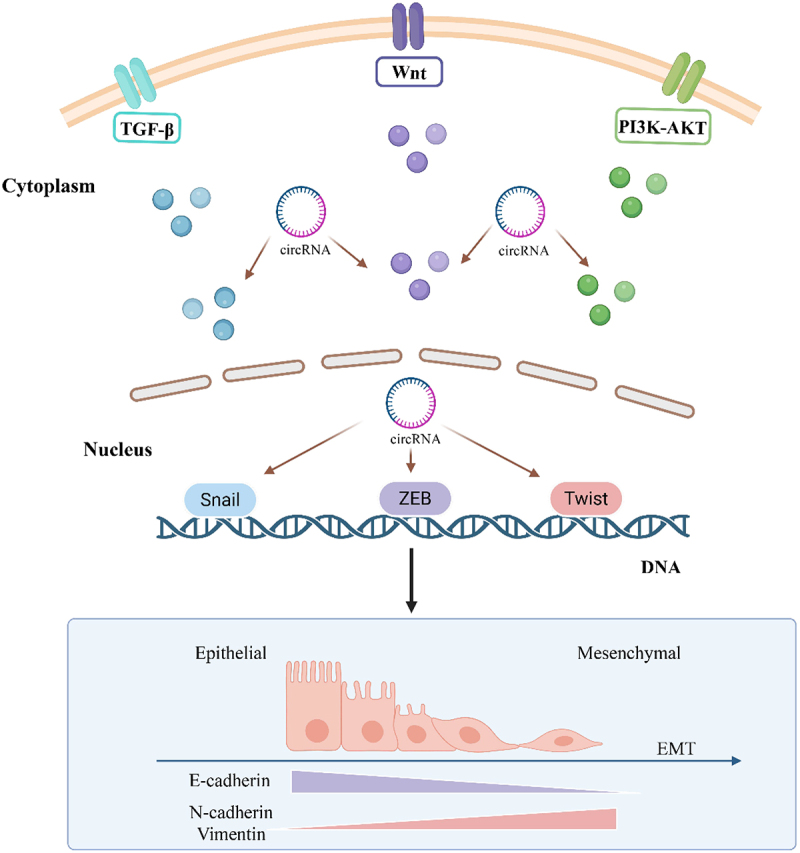
Comprehensive regulatory network of circRNA-mediated EMT regulation. In the cytoplasm, circRNAs directly or indirectly promote or inhibit EMT by interacting with key signaling molecules in the TGF-β, Wnt/β-catenin, and PI3K/AKT pathways. In the nucleus, circRNAs facilitate or suppress EMT by modulating EMT-transcription factors including Snail, ZEB, and Twist families. During EMT progression, epithelial cells undergo phenotypic transition to mesenchymal cells, characterized by downregulation of epithelial markers (E-cadherin) and upregulation of mesenchymal markers (N-cadherin and vimentin).

A reciprocal regulatory relationship exists between circRNAs and EMT-TFs, forming a complex, integrated network. CircRNAs not only function as upstream regulatory factors of EMT-TFs but can also be regulated by EMT-TFs, thereby acting on EMT. In hepatocellular carcinoma, TWIST1 binds to the Cul2 promoter to activate Cul2 transcription, leading to production of circ-10720 through back-splicing [60]. Circ-10720 subsequently functions as a key downstream effector and an integrative ceRNA node, sequestering multiple microRNAs (miR-1246, miR-578, and miR-490-5p) that normally target vimentin mRNA, thereby de-repressing vimentin expression and promoting EMT [60]. Cul2 is a tumour suppressor protein that degrades ubiquitinated HIFα and regulates the cell cycle [105,106]. In experiments, since circ-10720 sponged miR-1246, miR-578, and miR-490-5p, which are primary miRNAs regulating vimentin, it is hypothesized that TWIST1 promotes circ-10720 production, thereby preventing multiple miRNAs that inhibit vimentin expression from functioning, thus promoting vimentin expression. Vimentin, as a mesenchymal marker protein, promotes EMT in HCC cells. A similar regulatory mechanism appears to be widespread in non-small cell lung cancer, where circ-10720 was observed to be upregulated in tumour tissues, and changes in vimentin levels regulate EMT, thus affecting migration and invasion [107]. These studies suggest that the regulation between circRNA and EMT-TFs is intricate, with functional interactions occurring both upstream and downstream, ultimately establishing a self-reinforcing circuit where circRNAs act not just as effectors but as core components sustaining the pro-EMT feedback loop. (Figure 4)

### 4.5. Other regulatory pathways

Beyond the classical signalling pathways intensively studied in the regulatory relationship between EMT and circRNA, researchers have examined additional regulatory routes.

CircRNA regulation of the VEGFA/VEGFR2 pathway represents a critical mechanism in cancer progression, where circRNAs act as upstream modulators of angiogenesis and EMT [108]. Vascular endothelial growth factor A (VEGFA) is a member of the growth factor family with angiogenic properties and is a key regulator of angiogenesis in cancer tumours [109]. In bladder cancer, circRNA-MYLK, functioning as a pivotal ceRNA hub, sponges miR-29a, which normally targets the VEGFA 3’-UTR to inhibit its transcription. The sequestration of miR-29a by this circRNA leads to increased VEGFA levels, promoting the phosphorylation of VEGFR2 and thereby activating the VEGFA/VEGFR2 pathway. This activation subsequently triggers the phosphorylation of the downstream RAS/ERK signalling pathway, a cascade orchestrated by the initial circRNA-mediated event, which ultimately induces EMT [110]. Thus, circRNA-MYLK serves as a central regulator that integrates angiogenic signalling with the EMT programme.

CircRNA-mediated regulation of the SOX4/EZH2 axis plays a key role in pancreatic cancer progression, as exemplified by the oncogenic function of circ_0001666. Circ_0001666 is overexpressed in pancreatic cancer samples, and its high expression is associated with poor patient prognosis. Functioning as a central ceRNA network hub, circ_0001666 can sponge miR-1251, which targets SOX4, thereby derepressing SOX4 expression [111]. SOX4 subsequently functions as a direct transcriptional activator of the EZH2 promoter, inducing EZH2 expression to enhance EMT in pancreatic cancer [112]. Therefore, circ_0001666 acts as the upstream master regulator that initiates the oncogenic cascade, driving EMT through the miR-1251/SOX4/EZH2 axis in PC tissues [113], thereby positioning this circRNA as a critical integrator of transcriptional and epigenetic control within the EMT network.

CircRNA sequestration of RNA-binding proteins such as HuR represents a direct post-transcriptional regulatory mechanism affecting EMT, as demonstrated in cervical cancer by circPABPN1. Derived from the PABPN1 gene, circPABPN1 serves as a central molecular decoy that can bind to and sequester HuR, an RNA-binding protein that regulates protein translation by binding to mRNA, thus acting as a sponge for HuR and inhibiting PABPN1 translation in cervical cancer cells [114]. Simultaneously, HuR can bind to the 3’-UTR of EMT transcription factor mRNA to increase Snail mRNA stability, causing increased Snail expression to promote EMT [115]. This indicates that circPABPN1, through its sequestration of HuR, orchestrates a dual regulatory function: suppressing PABPN1 translation while indirectly enhancing Snail stability, thereby positioning itself as a key upstream regulator that coordinately promotes EMT-related processes. (Table 1)

**Table 1.** Representative CircRNAs regulating EMT in different cancer types.

molecular mechanism	circRNA	Cancer type	Targets\effectors	Biological function	References
miRNA sponge	hsa_circ_0088036	NSCLC	miR-1343-3p	Promotes EMT	[62]
	circPTK2	NSCLC	miR-429/miR-200b-3p	Inhibits EMT	[67]
	circUHRF1	OSCC	miR-526b-5p	Promotes EMT	[72]
	circANKS1B	BC	miR-148a\miR-152-3p	Promotes EMT	[74]
	hsa_circ_0009361	CRC	miR-582	Inhibits EMT	[77]
	circRNA_100367	ESCC	miR-217	Reduced resistance to EMT	[78]
	circ_0026344	CRC	miR-183	Inhibits EMT	[79]
	circRNA_0082835	SKCM	miR-429	Promotes EMT	[83]
	circGRAMD1B	LUAD	miR-4428	Promotes EMT	[92]
	circRNAHIPK3	OSCC	miR-637	Promotes EMT	[95]
	circPIP5K1A	Glioma	miR-515-5p	Promotes EMT	[96]
	circRNA_0084043	SKCM	miR-153-3p	Promotes EMT	[82]
	circPRMT5	UCB	miR-30c	Promotes EMT	[102]
	circPCNXL2	ccRCC	miR-153	Promotes EMT	[104]
	circ-10720	HCC	miR-1246\miR-578\miR-490-5p	Promotes EMT	[107]
	circRNA-MYLK	BC	miR-29a	Promotes EMT	[110]
	circ_0001666	PC	miR-1251	Promotes EMT	[111]
Protein decoy	circGSK3β	ESCC	GSK3β	Promotes β-Catenin activity	[85]
	circNDST1	TC	CSNK2A1	Promotes EMT	[91]
	circPABPN1	CCA	HuR	Promotes EMT	[114]
Coding peptides	circβ-catenin	CA	GSK3β	Promotes EMT	[86]

## 5. CircRNAs as diagnostic biomarkers and therapeutic targets

### 5.1. Potential of circRNA as biomarkers

Compared with linear RNAs, circRNAs are structurally stable and are not degraded by RNA exonucleases, exhibiting stable expression patterns [116]. This intrinsic stability, coupled with their disease-specific dysregulation, forms the cornerstone of their utility as biomarkers in oncology. Critically, a growing body of clinical cohort studies has begun to translate these theoretical advantages into validated performance metrics, directly supporting their diagnostic and prognostic potential.

The promise of circRNAs as effective biomarkers is underscored by several key attributes, now substantiated by initial clinical evidence: (1) Their central role in pathological processes like EMT and metastasis is reflected in patient outcomes. For instance, clinical studies have validated that specific circRNAs are independent prognostic factors. As shown in Table 2, the aberrantly high expression of hsa_circ_0006006 in NSCLC patient serum is significantly associated with poor survival prognosis and cisplatin resistance [117], while in HCC, low expression of hsa_circ_0003570 correlates with advanced tumour stage and serves as an independent favourable prognostic factor [120]. This positions circRNA levels as dynamic indicators of disease aggression. (2) CircRNAs exhibit high tissue- and disease-specificity [125]. This specificity is crucial for diagnostic accuracy, as demonstrated by studies where circRNA signatures successfully distinguish cancer patients from healthy individuals. (3) Their abundance in stable, accessible body fluids like plasma and saliva [126,127] and their enrichment within exosomes [128] facilitate non-invasive ‘liquid biopsy’ applications. Researchers have successfully differentiated healthy populations from colon cancer patients by analysing serum exosomes [129].

**Table 2.** Clinical cohort studies of representative circular RNAs in different types of cancer.

circRNA	Cancer type	Patient cohort size	Association with clinical staging	Prognostic value	References
hsa_circ_0006006	NSCLC	52	The study did not clearly report the association with TNM staging	Abnormally high expression is significantly associated with poor survival prognosis and cisplatin resistance.	[117]
circ_0004592	GC	100	The level of expression is significantly associated with tumour differentiation, depth of invasion, and lymph node metastasis.	It is a potential diagnostic biomarker with an AUC value that has high sensitivity and specificity.	[118]
hsa_circ_0008621	CRC	257	Serum levels were significantly elevated in late-stage samples	High serum expression is an independent prognostic factor	[119]
hsa_circ_0003570	HCC	121	Low expression is associated with advanced TNM stage, larger tumours, and vascular invasion.	High expression is an independent factor for a favourable prognosis	[120]
hsa_circ_0057105	RCC	130	Expression is associated with advanced TNM staging	Independent prognostic factors for poor survival	[121]
hsa_circ_0002938	ESCC	50	High expression is positively correlated with TNM staging, tumour invasion extent, and lymph node metastasis.	Can be considered a potential predictive biomarker and therapeutic target for treating ESCC	[122]
hsa_circ_0087378	BC	23	Validated as downregulated in ER-positive breast cancer	It is a candidate target for the prospect of ER treatment	[123]
circ_PVT1	CRC	296	High expression is associated with TNM stage, tumour size, and lymph node metastasis.	Patients with high expression have a poorer prognosis	[124]

Systematic evaluation of diagnostic/prognostic performance metrics is emerging. Beyond mere detection, studies are quantifying the clinical value of circRNAs using standard biomarker metrics. For example, plasma circ_0004592 shows promise for the early detection of gastric cancer (GC), with its diagnostic power characterized by a receiver operating characteristic (ROC) curve analysis yielding an area under the curve (AUC) value that indicates high sensitivity and specificity [118]. In colorectal cancer (CRC), high serum levels of hsa_circ_0008621 constitute an independent prognostic factor [119], and in ESCC, hsa_circ_0002938 is not only correlated with advanced stage but is considered a potential predictive biomarker [122]. These studies move beyond association to establish quantifiable performance criteria.

Comparisons with established clinical biomarkers highlight both potential and the path forward. While traditional protein markers (e.g. CEA for CRC [130], CA-19–9 for pancreatic cancer [131]) are widely used, they often suffer from limited sensitivity or specificity. Preliminary comparative analyses suggest that certain circRNAs, either alone or in panels, may outperform or complement these conventional markers. In gastric cancer, for instance, the correlation of reduced plasma circKIAA1244 and elevated hsa_circ_0000467 with patient prognosis [132] suggests additive value. The ultimate clinical utility will likely reside in multi-analyte models that integrate circRNAs with existing markers (e.g. proteins, ctDNA) and clinical parameters, potentially enabling earlier detection, more accurate risk stratification, and real-time monitoring of therapeutic response. However, large-scale, prospective, multi-centre validation studies are imperative to rigorously establish their clinical superiority, standardize detection protocols, and define clear diagnostic cut-off values before routine clinical adoption.

### 5.2. Therapeutic strategies targeting circRNAs

Numerous studies have demonstrated that the knockdown or elevation of circRNAs has marked effects on the EMT pathway in cancer development. The primary existing therapeutic strategies involve targeted regulation of the cancer process through modulating target circRNA expression. The main methods include [133–135]: (1) CircRNA overexpression strategies: CircRNA vectors and techniques for synthetic-mediated circRNA overexpression can be employed to increase tumour-suppressive circRNA levels. (2) CircRNA delivery approaches: Exosome or nanoparticle-mediated circRNA delivery strategies can be used to introduce therapeutic circRNAs into target cells. (3) CircRNA knockdown strategies: These include approaches mediated by siRNA, short hairpin RNA, antisense oligonucleotides, and CRISPR/Cas9 systems to reduce oncogenic circRNA expression.

**Figure 5.**
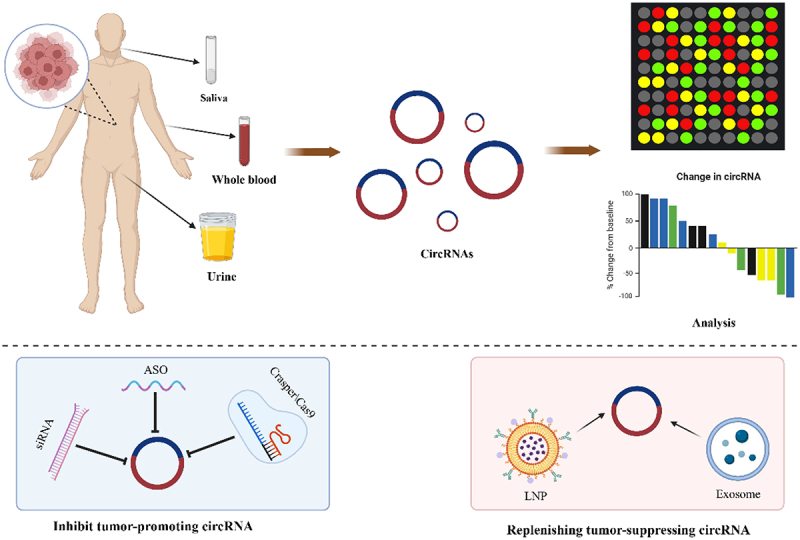
CircRNAs as diagnostic biomarkers and therapeutic targets in cancer. Upper panel: CircRNAs can be detected in body fluids including saliva, blood, and urine. Analysis of circRNA expression profiles enables rapid identification of tumour type and stage. Lower panel: therapeutic strategies targeting circRNAs include: (1) inhibiting oncogenic circRNAs using siRNA, antisense oligonucleotides (ASO), or CRISPR/Cas9 systems; (2) delivering tumour-suppressive circRNAs via lipid nanoparticles (LNPs) or exosomes to restore their expression in cancer cells.

Although circRNA-based cancer therapies have opened new avenues for oncology treatment, their clinical translation faces a series of complex challenges that must be urgently addressed. These challenges are first reflected in the delivery aspect: traditional delivery vectors, such as cationic liposomes, encounter bottlenecks when delivering molecules [136]. For instance, the limitations of vector design are highlighted by a study in which an attempt to achieve targeted siRNA delivery to a prostate cancer mouse model resulted in less than 6% tumour accumulation efficiency [137]. While emerging materials such as gold nanoparticles show promise in drug delivery, the in vivo safety and biodistribution profiles for applications involving circular RNAs still require comprehensive evaluation [138,139]. Secondly, at the specificity level, rational design is necessary to achieve precise targeting. For example, one study demonstrated that binding to the AAT promoter forms an apoptosis factor Bax-based tumour-killing switch, thereby enhancing hepatocyte specificity and enabling activation exclusively in specific hepatocellular carcinoma cells [140]. Furthermore, the inherent high stability of circRNA presents a dual challenge. Compared to mRNA, circRNA enables superior stability and sustained gene expression in cells. However, the excessively long half-life poses safety concerns for circRNA. In one study, researchers developed a ‘circRNA switch’ capable of sensing intracellular RNA or proteins to control circRNA-encoded protein expression, thereby ensuring the absence of severe cytotoxicity and immunogenicity, while remaining responsive to target miRNAs or proteins [141]. Finally, the risk of off-target effects is also substantial. Given the global regulatory capacity of circRNAs, the fact that a single circRNA can sponge multiple miRNAs suggests that its role as a ‘molecular sponge’ may disrupt miRNA networks globally [142]. Therefore, translating circRNA into a viable therapy is by no means straightforward. It must rely on the systematic integration of intelligent vector engineering, conditional expression design, and dynamic safety regulation to ultimately realize its therapeutic potential. (Figure 5) (Table 2)

## 6. Conclusions and future perspectives

In 2022, there were about 20 million new cancer cases worldwide [143], with 4,820,000 new cancers occurring in China [144], accounting for 24.1% of the global total. The International Union Against Cancer believes that one-third of cancers can be prevented, one-third can be cured if diagnosed early, and for the remaining one-third, pain can be alleviated and life prolonged, leading to the concept of tertiary prevention of malignant tumours. Since cancer represents a major threat and challenge to human health, understanding its pathogenesis, treatment, and prevention is one of the most critical tasks for researchers [145].

It is now evident that EMT in cancer metastasis is closely related to circRNA expression and function. CircRNAs are involved in vital EMT processes, such as initiation, maintenance of stem-like properties, and promotion of invasion. This suggests that circRNA dysregulation may be an essential driver of EMT and that in-depth study of these mechanisms may lead to novel therapeutic solutions [43].

The central position of circRNAs in cellular pathways and their close correlation with the upstream and downstream expression of EMT-TFs illustrates the importance of studying circRNAs for understanding carcinogenesis, metastasis, and EMT mechanisms. Additionally, as cancer biomarkers, circRNAs have unique advantages including stability, tissue specificity, and accessibility in body fluids [146]. CircRNA-based biological treatments may become a novel approach and promising direction for cancer therapy.

However, circRNA-based therapeutic approaches face numerous challenges in the transition from basic research to clinical application. First, there are limitations in research models. In vitro cell models serve as the foundation for circRNA functional studies, but standard cell lines lose many endogenous circRNAs during long-term culture [147], and their expression patterns differ from those in primary tumours. This may lead to functional experimental results based on such models not reflecting the actual in vivo situation. Animal models are crucial bridges for translating circRNA research into clinical practice; however, due to the significant species specificity of circRNAs, their distribution and function may differ markedly between humans and experimental animals [148]. This makes it difficult to replicate circRNA distribution observed in mouse models in humans, and the functions of the same circRNA may vary between species [149], hindering translation from basic research to clinical applications.

Second, methodological limitations significantly hinder their translation from basic research to clinical practice. A key feature of circRNAs is back-splicing, making it essential to confirm that detected signals indeed originate from circRNAs formed through back-splicing and to distinguish different splice isoforms from the same gene locus. Traditional methods based on back-splicing junction detection, such as RNA-seq and RT-qPCR, often struggle to precisely differentiate and accurately identify distinct isoforms, which affects subsequent functional research and the specificity evaluation of biomarkers. Following this is the issue of bias introduced by RNase R treatment. CircRNAs are present in various sample types such as tissues, plasma, and exosomes [150], yet standardized extraction protocols are lacking, leading to difficulties in comparing data across different studies. RNase R digestion is a commonly used method for enriching circRNAs [151], but subtle variations in treatment conditions – such as enzyme concentration – can significantly impact outcomes: excessive enzyme may degrade circRNAs, while insufficient amounts may leave residual linear RNAs, resulting in inaccurate detection results. Challenges also persist in circRNA detection and data analysis methodologies, as different enrichment strategies, sequencing approaches, and identification algorithms can yield substantially divergent results [152]. A comparative study revealed that the number of circRNAs identified in the same cell line could vary by up to 42-fold depending on the tool used [153]. Moreover, qPCR quantification of circRNAs currently lacks universally accepted and reliable internal reference genes comparable to GAPDH or β-actin used for mRNAs. At the same time, the entire workflow – from sample collection and preservation to RNA extraction and reverse transcription – lacks unified standards, making data across laboratories difficult to compare and integrate. This is a key obstacle preventing circRNAs from becoming clinically reliable biomarkers.

Finally, there are challenges in clinical application. Although circRNAs show great promise as biomarkers for cancer metastasis, their accurate detection remains difficult with current technologies. Although circRNAs are structurally stable, their half-life and clearance mechanisms in the blood are not yet fully understood, which affects their reliability as dynamic monitoring indicators. Additionally, many circRNAs that are aberrantly expressed in cancer are also involved in other pathological processes, such as cardiovascular diseases and Alzheimer’s disease [154], leading to insufficient diagnostic specificity.

To systematically overcome the translational bottlenecks from basic research to clinical application for circRNAs, future efforts must integrate cutting-edge technologies, innovative models, and intelligent design strategies through a focused and convergent approach along three core pathways.

First, on the technological and standardization pathway, it is essential to transcend the current limitations of short-read sequencing and RNase R enrichment [155]. The solution involves the development and widespread adoption of single-molecule real-time full-length sequencing technologies, such as those offered by PacBio Sequel IIIe or Oxford Nanopore platforms. These technologies directly decipher the complete circular sequence and internal modifications of circRNAs, thereby providing a fundamental resolution to the challenges of authenticating back-splicing junctions and precisely characterizing isoforms [156]. Concurrently, establishing a globally collaborative reference database and algorithmic benchmarking platform is crucial. Initiatives like a proposed Circ-omics Standardization. Initiative could furnish the community with unified reference materials, standardized protocols, and open-source analytical tools. This framework would enable the systematic calibration and performance assessment of laboratory workflows – from library preparation and sequencing to data analysis – ultimately fostering reproducible and comparable quantitative standards across the field.

Second, on the research and validation model pathway, there is a pressing need to develop experimental systems that more accurately mirror the intricate reality of human tumours. The central strategy is to advance a four-dimensional functional screening platform based on patient-derived tumour organoids integrated with organ-on-a-chip technology. This involves coupling organoids generated from fresh patient tumour tissues with sophisticated microfluidic systems that incorporate essential stromal components like immune cells and fibroblasts [157]. Conducting high-throughput functional screenings on such a platform allows for the identification of circRNAs that genuinely drive tumour progression within a context that preserves native microenvironmental cues, cellular heterogeneity, and dynamic growth patterns [158]. To validate these findings without artefact, endogenous circRNA expression should be precisely modulated directly within this system using advanced in situ editing tools like CRISPR-dCas13 [159], moving beyond conventional overexpression models that may yield misleading results.

Finally, the clinical application design pathway necessitates a paradigm shift from mere biomarker discovery towards the creation of intelligent therapeutics. For diagnostic advancement, the goal is to construct a dynamic, multi-omics-integrated liquid biopsy monitoring model. This approach surpasses the simple combination of a few markers by longitudinally analysing patient plasma samples throughout therapy. It integrates temporal data from circRNA expression profiles, circulating tumour DNA mutation spectra, proteomics, and exosome content. Leveraging advanced temporal deep learning models, such as graph neural networks, this model can unravel complex biosignature networks capable of predicting therapeutic response, the emergence of drug resistance, or early signs of relapse [160]. For therapeutic innovation, the field is expanding along two complementary axes. One axis focuses on engineering modular sense-compute-execute closed-loop circRNA circuits. An example is a designer circRNA encoding a tandem of functional modules: a sensing module featuring a peptide linker specifically cleaved by proteases overactive in the tumour microenvironment; a logic computation module housing a riboswitch activated only by a precise combination of miRNA signals; and an execution-and-self-destruct module that produces a therapeutic protein but contains degradation elements in its 3’UTR triggered by miRNAs abundant in normal tissues. This design ensures spatially and contextually restricted activation followed by safe elimination. The other axis leverages drug repurposing strategies, such as repurposing drugs as GLP-1 based therapy or targeting 20S proteasomes and giving various natural compounds as hinokitiol as prophylactic with immuno-modulatory effect with positive impact on cancer [161]. Together, these strategies form a multidimensional and potentially synergistic new framework for cancer treatment.

In conclusion, the clinical translation of circRNAs represents a collaborative revolution, demanding concerted contributions from nanotechnology for precision delivery, synthetic biology for intelligent circuit design, bioinformatics for complex data deconvolution, and clinical medicine for pioneering trial design. Only through such a deeply integrated and engineering-minded endeavour can the vast theoretical promise of circRNAs be transformed into tangible diagnostic and therapeutic tools for patients.

## Abbreviations

circRNA: Circular RNA
EMT: epithelial-mesenchymal transition
TGF-β: transforming growth factor-β;
EMT-TFs: EMT-activated transcription factors
ceRNA: competing endogenous RNA
ncRNAs: non-coding RNAs;
miRNA: microRNA;
lncRNA: long non-coding RNA
CiRNAs: intronic circRNAs
ElcRNAs: exon-intron circRNAs
IRES: internal ribosome entry sites
m6A: N6-methyladenosine;
CA: cancel;
NSCLC: non-small cell lung cancer;
OSCC: oral squamous cell carcinoma;
BC: breast cancer;
CRC: colorectal cancer
ESCC: oesophageal squamous cell carcinoma
SKCM: skin cutaneous melanoma
LUAD: lung adenocarcinoma
UCB: urothelial carcinoma of the bladder
ccRCC: clear cell renal cell carcinoma
HCC: hepatocellular carcinoma
PC: prostate cancer
TC: thyroid carcinoma
CCA: cervical cancer.

## Author information

*Authors and Affiliations*.

Joint Lab of Reproductive Medicine of SCU-CUHK, Lab of Reproductive genetics and Epigenetics, Department of Obstetrics/Gynaecology, Key Laboratory of Birth Defects and Related Disease of Women and Children of MOE, West China Second University Hospital, Sichuan University, 610,041, Chengdu, China.

Zekai Lv.

Department of Critical Care Medicine, Frontiers Science Center for Disease-relatea Molecular Network, State Key Laboratory of Biotherapy and Cancer Center, WestChina Hospital, Sichuan University, Chengdu 61,004 l, China.

Guang Xie.

Joint Lab of Reproductive Medicine of SCU-CUHK, Lab of Reproductive genetics and Epigenetics, Department of Obstetrics/Gynaecology, Key Laboratory of Birth Defects and Related Disease of Women and Children of MOE, West China Second University Hospital, Sichuan University, 610,041, Chengdu, China.

Wengming Xu.

## Declarations

No datasets were generated or analysed during the current study.

## References

[1] Atta H, Alzahaby N, Hamdy NM, et al. New trends in synthetic drugs and natural products targeting 20S proteasomes in cancers. Bioorg Chem. 2023;133:106427. doi: 10.1016/j.bioorg.2023.106427 36841046

[2] Chiang YF, Huang KC, Chen HY, et al. Hinokitiol inhibits breast cancer cells in vitro stemness-progression and self-renewal with apoptosis and autophagy modulation via the CD44/Nanog/SOX2/Oct4 pathway. Int J Mol Sci. 2024;25(7):25. doi: 10.3390/ijms25073904 PMC11011552 38612715

[3] Mani K, Deng D, Lin C, et al. Causes of death among people living with metastatic cancer. Nat Commun. 2024;15(1):1519. doi: 10.1038/s41467-024-45307-x 38374318 PMC10876661

[4] Chen T, You Y, Jiang H, et al. Epithelial–mesenchymal transition (EMT): a biological process in the development, stem cell differentiation, and tumorigenesis. J Cell Physiol. 2017;232(12):3261–3272. doi: 10.1002/jcp.25797 28079253 PMC5507753

[5] Sokolov D, Sharda N, Banerjee A, et al. Differential signaling pathways in medulloblastoma: nano-biomedicine targeting non-coding epigenetics to improve current and future therapeutics. Curr Pharm Des. 2024;30(1):31–47. doi: 10.2174/0113816128277350231219062154 38151840

[6] Eldash S, Sanad EF, Nada D, et al. The intergenic type LncRNA (LINC RNA) faces in cancer with in silico scope and a directed lens to LINC00511: a step toward ncRNA precision. ncRNA. 2023;9(5):9. doi: 10.3390/ncrna9050058 37888204 PMC10610215

[7] Trusolino L, Comoglio PM. Scatter-factor and semaphorin receptors: cell signalling for invasive growth. Nat Rev Cancer. 2002;2(4):289–300. doi: 10.1038/nrc779 12001990

[8] Chaffer CL, Weinberg RA. A perspective on cancer cell metastasis. Science. 2011;331(6024):1559–1564. doi: 10.1126/science.1203543 21436443

[9] Nieto MA, Huang R-J, Jackson RA, et al. EMT: 2016. Cell. 2016;166(1):21–45. doi: 10.1016/j.cell.2016.06.028 27368099

[10] Lamouille S, Xu J, Derynck R. Molecular mechanisms of epithelial–mesenchymal transition. Nat Rev Mol Cell Biol. 2014;15(3):178–196. doi: 10.1038/nrm3758 24556840 PMC4240281

[11] Yang J, Antin P, Berx G, et al. Guidelines and definitions for research on epithelial–mesenchymal transition. Nat Rev Mol Cell Biol. 2020;21(6):341–352. doi: 10.1038/s41580-020-0237-9 32300252 PMC7250738

[12] Fazilaty H, Rago L, Kass Youssef K, et al. A gene regulatory network to control EMT programs in development and disease. Nat Commun. 2019;10(1):5115. doi: 10.1038/s41467-019-13091-8 31712603 PMC6848104

[13] Barrallo-Gimeno A, Nieto MA. The snail genes as inducers of cell movement and survival: implications in development and cancer. Development. 2005;132(14):3151–3161. doi: 10.1242/dev.01907 15983400

[14] Thiery JP, Acloque H, Huang RYJ, et al. Epithelial-mesenchymal transitions in development and disease. Cell. 2009;139(5):871–890. doi: 10.1016/j.cell.2009.11.007 19945376

[15] Saitoh M. Transcriptional regulation of EMT transcription factors in cancer. Semin Cancer Biol. 2023;97:21–29. doi: 10.1016/j.semcancer.2023.10.001 37802266

[16] Hsu DS, Wang HJ, Tai SK, et al. Acetylation of snail modulates the cytokinome of cancer cells to enhance the recruitment of macrophages. Cancer Cell. 2014;26(4):534–548. doi: 10.1016/j.ccell.2014.09.002 25314079

[17] Hamdy NM, El-Sisi MG, Ibrahim SM, et al. In silico analysis and comprehensive review of circular-RNA regulatory roles in breast diseases; a step-toward non-coding RNA precision. Pathol Res Pract. 2024;263:155651. doi: 10.1016/j.prp.2024.155651 39454476

[18] Zhang X, Wu H, Hong X, et al. Circular RNA: from non-coding regulators to functional protein encoders. Pharm Sci Adv. 2025;3:100085. doi: 10.1016/j.pscia.2025.100085 41550639 PMC12710065

[19] Youness RA, Hassan HA, Abaza T, et al. A comprehensive insight and in silico analysis of circRNAs in hepatocellular carcinoma: a step toward ncRNA-based precision medicine. Cells. 2024;13(15):13. doi: 10.3390/cells13151245 PMC11312109 39120276

[20] Tu C, He J, Qi L, et al. Emerging landscape of circular RNAs as biomarkers and pivotal regulators in osteosarcoma. J Cell Physiol. 2020;235(12):9037–9058. doi: 10.1002/jcp.29754 32452026

[21] Chen YT, Tsai HJ, Kan CH, et al. Noncanonical formation of SNX5 gene-derived circular RNA regulates cancer growth. Cell Death Dis. 2024;15(8):599. doi: 10.1038/s41419-024-06980-4 39155279 PMC11330969

[22] O’Leary E, Jiang Y, Kristensen LS, et al. The therapeutic potential of circular RNAs. Nat Rev Genet. 2025;26(4):230–244. doi: 10.1038/s41576-024-00806-x 39789148

[23] Rajan AAN, Hutchins EJ. Post-transcriptional regulation as a conserved driver of neural crest and cancer-cell migration. Curr Opin Cell Biol. 2024;89:102400. doi: 10.1016/j.ceb.2024.102400 39032482 PMC11346372

[24] Samad MA, Ahmad I, Suhail M, et al. Role of circular RNAs in regulating tumor microenvironment, epithelial mesenchymal transition, and resistance to cancer therapy. Crit Rev Oncol Hematol. 2025;216:104942. doi: 10.1016/j.critrevonc.2025.104942 40953760

[25] Wang C, Tan S, Liu WR, et al. RNA-seq profiling of circular rNA in human lung adenocarcinoma and squamous cell carcinoma. Mol Cancer. 2019;18(1):134. doi: 10.1186/s12943-019-1061-8 31484581 PMC6724331

[26] Wu Z, Yu X, Zhang S, et al. Mechanism underlying circRNA dysregulation in the TME of digestive system cancer. Front Immunol. 2022;13:951561. doi: 10.3389/fimmu.2022.951561 36238299 PMC9550895

[27] Conn SJ, Pillman KA, Toubia J, et al. The RNA binding protein quaking regulates formation of circRNAs. Cell. 2015;160(6):1125–1134. doi: 10.1016/j.cell.2015.02.014 25768908

[28] Ashrafizadeh M, Dai J, Torabian P, et al. Circular RNAs in EMT-driven metastasis regulation: modulation of cancer cell plasticity, tumorigenesis and therapy resistance. Cell Mol Life Sci. 2024;81(1):214. doi: 10.1007/s00018-024-05236-w 38733529 PMC11088560

[29] Sahib AS, Fawzi A, Zabibah RS, et al. MiRNA/epithelial-mesenchymal axis (EMT) axis as a key player in cancer progression and metastasis: a focus on gastric and bladder cancers. Cell Signal. 2023;112:110881. doi: 10.1016/j.cellsig.2023.110881 37666286

[30] Guo D, Sheng K, Zhang Q, et al. Single-cell transcriptomic analysis reveals the landscape of epithelial-mesenchymal transition molecular heterogeneity in esophageal squamous cell carcinoma. Cancer Lett. 2024;587:216723. doi: 10.1016/j.canlet.2024.216723 38342234

[31] Madhavan S, Nagarajan S. GRP78 and next generation cancer hallmarks: an underexplored molecular target in cancer chemoprevention research. Biochimie. 2020;175:69–76. doi: 10.1016/j.biochi.2020.05.005 32422159

[32] Chen Y, Guo S, Lu D, et al. Circular RNAs as promising biomarkers for human diseases: an update for 2018–2025. Genes Dis. 2026;2026:102043. doi: 10.1016/j.gendis.2026.102043

[33] Hama Faraj GS, Hussen BM, Abdullah SR, et al. Advanced approaches of the use of circRnas as a replacement for cancer therapy. Noncoding RNA Res. 2024;9(3):811–830. doi: 10.1016/j.ncrna.2024.03.012 38590433 PMC10999493

[34] Kim J. Circular RNAs: novel players in cancer mechanisms and therapeutic strategies. Int J Mol Sci. 2024;25(18):10121. doi: 10.3390/ijms251810121 39337606 PMC11432211

[35] Gundamaraju R, Lu W, Paul MK, et al. Autophagy and EMT in cancer and metastasis: who controls whom? Biochim Biophys Acta Mol Basis Dis. 2022;1868(9):166431. doi: 10.1016/j.bbadis.2022.166431 35533903

[36] Kim BN, Ahn DH, Kang N, et al. TGF-β induced EMT and stemness characteristics are associated with epigenetic regulation in lung cancer. Sci Rep. 2020;10(1):10597. doi: 10.1038/s41598-020-67325-7 32606331 PMC7326979

[37] Dong A, Blanpain C. Identification, functional insights and therapeutic targeting of EMT tumour states. Nat Rev Cancer. 2025;26(1):8–26. doi: 10.1038/s41568-025-00873-0 40999060

[38] Su Z, Yang Z, Xu Y, et al. Apoptosis, autophagy, necroptosis, and cancer metastasis. Mol Cancer. 2015;14(1):48. doi: 10.1186/s12943-015-0321-5 25743109 PMC4343053

[39] Xia J, Tian Y, Shao Z, et al. Malat1-miR-30c-5p-CTGF/ATG5 axis regulates silica-induced experimental silicosis by mediating EMT in alveolar epithelial cells. Ecotoxicol Environ Saf. 2023;249:114392. doi: 10.1016/j.ecoenv.2022.114392 36508811

[40] Song H, Zhao Z, Ma L, et al. MiR-3653 blocks autophagy to inhibit epithelial-mesenchymal transition in breast cancer cells by targeting the autophagy-regulatory genes ATG12 and AMBRA1. Chin Med J (Engl). 2023;136(17):2086–2100. doi: 10.1097/CM9.0000000000002569 37464439 PMC10476840

[41] Fedorova O, Daks A, Parfenyev S, et al. Zeb1-mediated autophagy enhances resistance of breast cancer cells to genotoxic drugs. Biochem Biophys Res Commun. 2022;589:29–34. doi: 10.1016/j.bbrc.2021.11.088 34883287

[42] Zhou Z, Zhang Y, Gao J, et al. Circular RNAs act as regulators of autophagy in cancer. Mol Ther Oncolytics. 2021;21:242–254. doi: 10.1016/j.omto.2021.04.007 34095462 PMC8142048

[43] Hashemi M, Khosroshahi EM, Daneii P, et al. Emerging roles of circRNA-miRNA networks in cancer development and therapeutic response. Noncoding RNA Res. 2025;10:98–115. doi: 10.1016/j.ncrna.2024.09.006 39351450 PMC11440256

[44] Bertheloot D, Latz E, Franklin BS. Necroptosis, pyroptosis and apoptosis: an intricate game of cell death. Cell Mol Immunol. 2021;18(5):1106–1121. doi: 10.1038/s41423-020-00630-3 33785842 PMC8008022

[45] Zhang C, Wang X, Miao Z, et al. Unleashing necroptosis: transforming the tumor immune microenvironment for cancer therapy. Biomed Pharmacother. 2026;195:118988. doi: 10.1016/j.biopha.2026.118988 41512557

[46] Peng F, Liao M, Qin R, et al. Regulated cell death (RCD) in cancer: key pathways and targeted therapies. Sig Transduct Target Ther. 2022;7(1):286. doi: 10.1038/s41392-022-01110-y PMC9376115 35963853

[47] Wang X, Wang B, Xie J, et al. Melatonin inhibits epithelial‑to‑mesenchymal transition in gastric cancer cells via attenuation of IL‑1β/NF‑κB/MMP2/MMP9 signaling. Int J Mol Med. 2018;42:2221–2228. doi: 10.3892/ijmm.2018.3788 30066836

[48] Lee H, Kim B, Park J, et al. Cancer stem cells: landscape, challenges and emerging therapeutic innovations. Sig Transduct Target Ther. 2025;10(1):248. doi: 10.1038/s41392-025-02360-2 PMC12322150 40759634

[49] Khan AQ, Hasan A, Mir SS, et al. Exploiting transcription factors to target EMT and cancer stem cells for tumor modulation and therapy. Semin Cancer Biol. 2024;100:1–16. doi: 10.1016/j.semcancer.2024.03.002 38503384

[50] Zhang X, Wei C, Li J, et al. MicroRNA-361-5p inhibits epithelial-to-mesenchymal transition of glioma cells through targeting Twist1. Oncol Rep. 2017;37(3):1849–1856. doi: 10.3892/or.2017.5406 28184914

[51] Sahoo S, Hari K, Jolly MK. Design principles of regulatory networks underlying epithelial mesenchymal plasticity in cancer cells. Curr Opin Cell Biol. 2025;92:102445. doi: 10.1016/j.ceb.2024.102445 39608060

[52] Huang Y, Mo W, Ding X, et al. Long non-coding RNAs in breast cancer stem cells. Med Oncol. 2023;40(6):177. doi: 10.1007/s12032-023-02046-1 37178429

[53] Chiang TW, Mai TL, Chuang TJ. CircMiMi: a stand-alone software for constructing circular RNA-microRNA-mRNA interactions across species. BMC Bioinf. 2022;23(1):164. doi: 10.1186/s12859-022-04692-0 PMC9074202 35524165

[54] Yang L, Wilusz JE, Chen LL. Biogenesis and regulatory roles of circular RNAs. Annu Rev Cell Dev Biol. 2022;38(1):263–289. doi: 10.1146/annurev-cellbio-120420-125117 35609906 PMC10119891

[55] Wei G, Zhu J, Hu HB, et al. Circular RNAs: promising biomarkers for cancer diagnosis and prognosis. Gene. 2021;771:145365. doi: 10.1016/j.gene.2020.145365 33346098

[56] Chen LL. The expanding regulatory mechanisms and cellular functions of circular RNAs. Nat Rev Mol Cell Biol. 2020;21(8):475–490. doi: 10.1038/s41580-020-0243-y 32366901

[57] Harsij Z, Mehrabi Z, Davoudi N. Exploring the complex dimensions of Alzheimer’s disease: the promising role of circular RNAs and their potential contributions. Gene. 2025;960:149549. doi: 10.1016/j.gene.2025.149549 40339771

[58] Sun M, Yang Y. Biological functions and applications of circRNAS—next generation of RNA-based therapy. J Mol Cell Biol. 2023;15(5):mjad031. doi: 10.1093/jmcb/mjad031 37147015 PMC10708935

[59] Muthuramalingam K, Cho M, Kim Y. Correction to: role of NADPH oxidase and its therapeutic intervention in TGF-β-mediated EMT progression: an in vitro analysis on HeLa cervical cancer cells. Appl Biol Chem. 2021;64(1):63. doi: 10.1186/s13765-021-00636-z

[60] Meng J, Chen S, Han JX, et al. Twist1 regulates vimentin through Cul2 circular RNA to promote EMT in hepatocellular carcinoma. Cancer Res. 2018;78(15):4150–4162. doi: 10.1158/0008-5472.CAN-17-3009 29844124

[61] Muñoz MD, de la Fuente N, Sánchez-Capelo A. TGF-β/Smad3 signalling modulates GABA neurotransmission: implications in Parkinson’s disease. Int J Mol Sci. 2020;21(2):21. doi: 10.3390/ijms21020590 PMC7013528 31963327

[62] Ge P, Chen X, Liu J, et al. Hsa_circ_0088036 promotes nonsmall cell lung cancer progression by regulating miR-1343-3p/Bcl-3 axis through TGFβ/Smad3/EMT signaling. Mol Carcinog. 2023;62(7):1073–1085. doi: 10.1002/mc.23547 37132942

[63] Zhang X, Wang S, Wang H, et al. Circular RNA circNRIP1 acts as a microRNA-149-5p sponge to promote gastric cancer progression via the AKT1/mTOR pathway. Mol Cancer. 2019;18(1):20. doi: 10.1186/s12943-018-0935-5 30717751 PMC6360801

[64] Li J, Ma M, Yang X, et al. Circular HER2 RNA positive triple negative breast cancer is sensitive to pertuzumab. Mol Cancer. 2020;19(1):142. doi: 10.1186/s12943-020-01259-6 32917240 PMC7488427

[65] Wang J, Li X, Lu L, et al. Circular RNA hsa_circ_0000567 can be used as a promising diagnostic biomarker for human colorectal cancer. J Clin Lab Anal. 2018;32(5):e22379. doi: 10.1002/jcla.22379 29333615 PMC6817158

[66] He W, Dorn DC, Erdjument-Bromage H, et al. Hematopoiesis controlled by distinct TIF1γ and Smad4 branches of the TGFβ pathway. Cell. 2006;125(5):929–941. doi: 10.1016/j.cell.2006.03.045 16751102

[67] Wang L, Tong X, Zhou Z, et al. Circular RNA hsa_circ_0008305 (circPTK2) inhibits TGF-β-induced epithelial-mesenchymal transition and metastasis by controlling TIF1γ in non-small cell lung cancer. Mol Cancer. 2018;17:140. doi: 10.1186/s12943-018-0889-7 30261900 PMC6161470

[68] Su Z, Sun Z, Wang Z, et al. TIF1γ inhibits lung adenocarcinoma EMT and metastasis by interacting with the TAF15/TBP complex. Cell Rep. 2022;41(3):111513. doi: 10.1016/j.celrep.2022.111513 36261009

[69] Tang S, Cai L, Wang Z, et al. Emerging roles of circular RNAs in the invasion and metastasis of head and neck cancer: possible functions and mechanisms. Cancer Innov. 2023;2(6):463–487. doi: 10.1002/cai2.50 38125767 PMC10730008

[70] Huang C, Shan G. What happens at or after transcription: insights into circRNA biogenesis and function. Transcription. 2015;6(4):61–64. doi: 10.1080/21541264.2015.1071301 26177684 PMC4802811

[71] El Marabti E, Younis I. The cancer spliceome: reprograming of alternative splicing in cancer. Front Mol Biosci. 2018;5:80. doi: 10.3389/fmolb.2018.00080 30246013 PMC6137424

[72] Zhao W, Cui Y, Liu L, et al. Splicing factor derived circular RNA circUHRF1 accelerates oral squamous cell carcinoma tumorigenesis via feedback loop. Cell Death Differ. 2020;27(3):919–933. doi: 10.1038/s41418-019-0423-5 31570856 PMC7206121

[73] Li S, Zhang S, Chen J. C-Myc induced upregulation of long non-coding RNA SNHG16 enhances progression and carcinogenesis in oral squamous cell carcinoma. Cancer Gene Ther. 2019;26(11–12):400–410. doi: 10.1038/s41417-018-0072-8 30607006

[74] Zeng K, He B, Yang BB, et al. The pro-metastasis effect of circANKS1B in breast cancer. Mol Cancer. 2018;17(1):160. doi: 10.1186/s12943-018-0914-x 30454010 PMC6240936

[75] Babaei G, Aziz SG, Jaghi NZZ. EMT, cancer stem cells and autophagy; the three main axes of metastasis. Biomed Pharmacother. 2021;133:110909. 33227701 10.1016/j.biopha.2020.110909

[76] Xu G, Zhang Z, Zhang L, et al. MiR-4326 promotes lung cancer cell proliferation through targeting tumor suppressor APC2. Mol Cell Biochem. 2018;443(1–2):151–157. doi: 10.1007/s11010-017-3219-2 29101731

[77] Geng Y, Zheng X, Hu W, et al. Hsa_circ_0009361 acts as the sponge of miR-582 to suppress colorectal cancer progression by regulating APC2 expression. Clin Sci (Lond). 2019;133(10):1197–1213. doi: 10.1042/CS20190286 31109967

[78] Liu J, Xue N, Guo Y, et al. Correction for: CircRNA_100367 regulated the radiation sensitivity of esophageal squamous cell carcinomas through miR-217/Wnt3 pathway. Aging (Albany NY). 2021;13(20):23868–23870. doi: 10.18632/aging.203664 34738919 PMC8580350

[79] Shen T, Cheng X, Liu X, et al. Circ_0026344 restrains metastasis of human colorectal cancer cells via miR-183. Artif Cells Nanomed Biotechnol. 2019;47(1):4038–4045. doi: 10.1080/21691401.2019.1669620 31608699

[80] Bi DP, Yin CH, Zhang XY, et al. Mir-183 functions as an oncogene by targeting ABCA1 in colon cancer. Oncol Rep. 2016;35(5):2873–2879. doi: 10.3892/or.2016.4631 26935154

[81] Yang X, Wang L, Wang Q, et al. Mir-183 inhibits osteosarcoma cell growth and invasion by regulating LRP6-Wnt/β-catenin signaling pathway. Biochem Biophys Res Commun. 2018;496(4):1197–1203. doi: 10.1016/j.bbrc.2018.01.170 29402412

[82] Luan W, Shi Y, Zhou Z, et al. Corrigendum to circRNA_0084043 promote malignant melanoma progression via miR-153-3p/Snail axis. Biochem Biophys Res Commun. 2022;587:168–169. doi: 10.1016/j.bbrc.2021.12.001 34895697

[83] Sun Y, Hou Z, Luo B, et al. Circular RNA circRNA_0082835 promotes progression and lymphatic metastasis of primary melanoma by sponging microRNA miRNA-429. Bioengineered. 2021;12(1):4159–4173. doi: 10.1080/21655979.2021.1953822 34288815 PMC8806410

[84] Sheng H, Guo YH, Cao DS, et al. MiR-429-5p attenuates the migration and invasion of malignant melanoma by targeting LIMK1. Eur Rev Med Pharmacol Sci. 2020;24(5):2625–2631. doi: 10.26355/eurrev_202003_20531 32196612

[85] Hu X, Wu D, He X, et al. CircGSK3β promotes metastasis in esophageal squamous cell carcinoma by augmenting β-catenin signaling. Mol Cancer. 2019;18(1):160. doi: 10.1186/s12943-019-1095-y 31722716 PMC6854808

[86] Liang WC, Wong CW, Liang PP, et al. Translation of the circular RNA circβ-catenin promotes liver cancer cell growth through activation of the Wnt pathway. Genome Biol. 2019;20(1):84. doi: 10.1186/s13059-019-1685-4 31027518 PMC6486691

[87] Liang W, Lai Y, Zhu M, et al. Combretastatin A4 regulates proliferation, migration, invasion, and apoptosis of thyroid cancer cells via PI3K/Akt signaling pathway. Med Sci Monit. 2016;22:4911–4917. doi: 10.12659/MSM.898545 27966519 PMC5179240

[88] Trembley JH, Wang G, Unger G, et al. Protein kinase CK2 in health and disease: CK2: a key player in cancer biology. Cell Mol Life Sci. 2009;66(11–12):1858–1867. doi: 10.1007/s00018-009-9154-y 19387548 PMC4385580

[89] Sato K, Padgaonkar AA, Baker SJ, et al. Simultaneous CK2/TNIK/DYRK1 inhibition by 108600 suppresses triple negative breast cancer stem cells and chemotherapy-resistant disease. Nat Commun. 2021;12(1):4671. doi: 10.1038/s41467-021-24878-z 34344863 PMC8333338

[90] Ponce DP, Yefi R, Cabello P, et al. CK2 functionally interacts with AKT/PKB to promote the β-catenin-dependent expression of survivin and enhance cell survival. Mol Cell Biochem. 2011;356(1–2):127–132. doi: 10.1007/s11010-011-0965-4 21735093

[91] Shu C, Wang S, Hu J, et al. CircNDST1 promotes papillary thyroid cancer progression via its interaction with CSNK2A1 to activate the PI3K–Akt pathway and epithelial–mesenchymal transition. J Endocrinol Invest. 2023;46(3):545–557. doi: 10.1007/s40618-022-01928-x 36306106 PMC9938055

[92] Liu X, Wang Y, Zhou G, et al. CircGRAMD1B contributes to migration, invasion and epithelial-mesenchymal transition of lung adenocarcinoma cells via modulating the expression of SOX4. Funct Integr Genomics. 2023;23(1):75. doi: 10.1007/s10142-023-00972-x 36867268

[93] Hanieh H, Ahmed EA, Vishnubalaji R, et al. SOX4 : epigenetic regulation and role in tumorigenesis. Semin Cancer Biol. 2020;67:91–104. doi: 10.1016/j.semcancer.2019.06.022 31271889

[94] Liang J, Li H, Han J, et al. Mex3a interacts with LAMA2 to promote lung adenocarcinoma metastasis via PI3K/AKT pathway. Cell Death Dis. 2020;11(8):614. doi: 10.1038/s41419-020-02858-3 32792503 PMC7427100

[95] Jiang W, Zhang C, Zhang X, et al. CircRNA HIPK3 promotes the progression of oral squamous cell carcinoma through upregulation of the NUPR1/PI3K/AKT pathway by sponging miR-637. Ann Transl Med. 2021;9(10):860. doi: 10.21037/atm-21-1908 34164494 PMC8184441

[96] Zheng K, Xie H, Wu W, et al. CircRNA PIP5K1A promotes the progression of glioma through upregulation of the TCF12/PI3K/AKT pathway by sponging miR-515-5p. Cancer Cell Int. 2021;21(1):27. doi: 10.1186/s12935-020-01699-6 33413401 PMC7789671

[97] Wang Z, Liu C. Upregulated hsa_circRNA_100269 inhibits the growth and metastasis of gastric cancer through inactivating PI3K/Akt axis. PLOS ONE. 2021;16(4):e0250603. doi: 10.1371/journal.pone.0250603 33901239 PMC8075232

[98] Jiang Y, Zhan H, Zhang Y, et al. ZIP4 promotes non-small cell lung cancer metastasis by activating snail-N-cadherin signaling axis. Cancer Lett. 2021;521:71–81. doi: 10.1016/j.canlet.2021.08.025 34450198

[99] Georgakopoulos-Soares I, Chartoumpekis DV, Kyriazopoulou V, et al. EMT factors and metabolic pathways in cancer. Front Oncol. 2020;10:499. doi: 10.3389/fonc.2020.00499 32318352 PMC7154126

[100] Brabletz S, Brabletz T. The ZEB/miR-200 feedback loop–a motor of cellular plasticity in development and cancer? EMBO Rep. 2010;11(9):670–677. doi: 10.1038/embor.2010.117 20706219 PMC2933868

[101] Zeng HF, Yan S, Wu SF. MicroRNA-153-3p suppresses cell proliferation and invasion by targeting SNAI1 in melanoma. Biochem Biophys Res Commun. 2017;487(1):140–145. doi: 10.1016/j.bbrc.2017.04.032 28400282

[102] Chen X, Chen RX, Wei WS, et al. Correction: PRMT5 circular RNA promotes metastasis of urothelial carcinoma of the bladder through sponging miR-30c to induce epithelial-mesenchymal transition. Clin Cancer Res. 2021;27(9):2664. doi: 10.1158/1078-0432.CCR-21-0936 33941547

[103] Chaffer CL, Marjanovic ND, Lee T, et al. Poised chromatin at the ZEB1 promoter enables breast cancer cell plasticity and enhances tumorigenicity. Cell. 2013;154(1):61–74. doi: 10.1016/j.cell.2013.06.005 23827675 PMC4015106

[104] Zhou B, Zheng P, Li Z, et al. CircPCNXL2 sponges miR-153 to promote the proliferation and invasion of renal cancer cells through upregulating ZEB2. Cell Cycle. 2018;17(23):2644–2654. doi: 10.1080/15384101.2018.1553354 30488762 PMC6300113

[105] Cai W, Yang H. The structure and regulation of Cullin 2 based E3 ubiquitin ligases and their biological functions. Cell Div. 2016;11(1):7. doi: 10.1186/s13008-016-0020-7 27222660 PMC4878042

[106] Maeda Y, Suzuki T, Pan X, et al. Cul2 is required for the activity of hypoxia-inducible factor and vasculogenesis. J Biol Chem. 2008;283(23):16084–16092. doi: 10.1074/jbc.M710223200 18372249 PMC2414293

[107] Martín J, Castellano JJ, Marrades RM, et al. Role of the epithelial-mesenchymal transition-related circular RNA, circ-10720, in non-small-cell lung cancer. Transl Lung Cancer Res. 2021;10(4):1804–1818. doi: 10.21037/tlcr-20-920 34012794 PMC8107756

[108] Koch S, Claesson-Welsh L. Signal transduction by vascular endothelial growth factor receptors. Cold Spring Harb Perspect Med. 2012;2(7):a006502. doi: 10.1101/cshperspect.a006502 22762016 PMC3385940

[109] Zhang YM, Miao ZM, Chen YP, et al. Ononin promotes radiosensitivity in lung cancer by inhibiting HIF-1α/VEGF pathway. Phytomedicine. 2024;125:155290. doi: 10.1016/j.phymed.2023.155290 38308918

[110] Zhong Z, Huang M, Lv M, et al. Corrigendum to “Circular RNA MYLK as a competing endogenous RNA promotes bladder cancer progression through modulating VEGFA/VEGFR2 signaling pathway” [Cancer Letter 403(2017) 305–317]. Cancer Lett. 2022;534:215631. doi: 10.1016/j.canlet.2022.215631 35300896

[111] Zhang R, Zhu W, Ma C, et al. Silencing of circRNA circ_0001666 represses EMT in pancreatic cancer through upregulating miR-1251 and downregulating SOX4. Front Mol Biosci. 2021;8:684866. doi: 10.3389/fmolb.2021.684866 34055896 PMC8155604

[112] Tiwari N, Tiwari VK, Waldmeier L, et al. Sox4 is a master regulator of epithelial-mesenchymal transition by controlling Ezh2 expression and epigenetic reprogramming. Cancer Cell. 2013;23(6):768–783. doi: 10.1016/j.ccr.2013.04.020 23764001

[113] Mody HR, Hung SW, AlSaggar M, et al. Inhibition of S-adenosylmethionine-dependent methyltransferase attenuates TGFβ1-induced EMT and metastasis in pancreatic cancer: putative roles of miR-663a and miR-4787-5p. Mol Cancer Res. 2016;14:1124–1135. 27624777 10.1158/1541-7786.MCR-16-0083 PMC5107158

[114] Abdelmohsen K, Panda AC, Munk R, et al. Identification of HuR target circular RNAs uncovers suppression of PABPN1 translation by CircPABPN1. RNA Biol. 2017;14(3):361–369. doi: 10.1080/15476286.2017.1279788 28080204 PMC5367248

[115] Dong P, Xu D, Xiong Y, et al. The expression, functions and mechanisms of circular RNAs in gynecological cancers. Cancers (Basel). 2020;12(6):12. doi: 10.3390/cancers12061472 PMC7352180 32512912

[116] Jeck WR, Sharpless NE. Detecting and characterizing circular RNAs. Nat Biotechnol. 2014;32(5):453–461. doi: 10.1038/nbt.2890 24811520 PMC4121655

[117] Ding M, Zhao J, Li X. Hsa_circ_0006006 is a potential biomarker for prognosis and cisplatin resistance in non-small cell lung cancer. Hereditas. 2025;162(1):32. doi: 10.1186/s41065-025-00392-w 40055838 PMC11889802

[118] Kong S, Xu YH, Zheng M, et al. Circ_0004592: an auxiliary diagnostic biomarker for gastric cancer. World J Gastrointest Oncol. 2024;16(6):2757–2768. doi: 10.4251/wjgo.v16.i6.2757 38994162 PMC11236232

[119] Zhou X, Wu L, Tian C. Overexpression of circular RNA hsa_circ_0008621 facilitates colorectal cancer progression and predicts poor prognosis. Ann Gastroenterol Surg. 2024;8(4):639–649. doi: 10.1002/ags3.12793 38957564 PMC11216790

[120] Jang SY, Kim G, Tak WY, et al. Circular noncoding RNA hsa_circ_0003570 as a prognostic biomarker for hepatocellular carcinoma. Genes (Basel). 2022;13(8):13. doi: 10.3390/genes13081484 PMC9407695 36011395

[121] Cen J, Liang Y, Feng Z, et al. Hsa_circ_0057105 modulates a balance of epithelial-mesenchymal transition and ferroptosis vulnerability in renal cell carcinoma. Clin Transl Med. 2023;13(8):e1339. doi: 10.1002/ctm2.1339 37496319 PMC10372385

[122] Li XP, Jia YL, Duan YQ, et al. Circular RNA hsa_circ_0002938 (circCRIM1) promotes the progression of esophageal squamous cell carcinoma by upregulating transcription factor 12. Neoplasma. 2023;70(01):145–157. doi: 10.4149/neo_2023_220823N857 36916930

[123] Yuan C, Zhou L, Zhang L, et al. Identification and integrated analysis of key differentially expressed circular RNAs in ER-positive subtype breast cancer. Epigenomics. 2019;11(3):297–321. doi: 10.2217/epi-2018-0147 30417652

[124] Mai S, Zhang Z, Mi W. Upregulation of circ_PVT1 and circ_001569 indicate unfavorable prognosis in colorectal cancer. Ann Clin Lab Sci. 2021;51(1):55–60. 33653781

[125] Zhou T, Xie X, Li M, et al. Rat bodymap transcriptomes reveal unique circular RNA features across tissue types and developmental stages. RNA. 2018;24(11):1443–1456. doi: 10.1261/rna.067132.118 30093490 PMC6191709

[126] Bahn JH, Zhang Q, Li F, et al. The landscape of microRNA, piwi-interacting RNA, and circular RNA in human saliva. Clin Chem. 2015;61(1):221–230. doi: 10.1373/clinchem.2014.230433 25376581 PMC4332885

[127] Memczak S, Papavasileiou P, Peters O, et al. Identification and characterization of circular RNAs as a new class of putative biomarkers in human blood. PLOS ONE. 2015;10(10):e0141214. doi: 10.1371/journal.pone.0141214 26485708 PMC4617279

[128] Zhang X, Yuan X, Shi H, et al. Exosomes in cancer: small particle, big player. J Hematol Oncol. 2015;8(1):83. doi: 10.1186/s13045-015-0181-x 26156517 PMC4496882

[129] Louis C, Desoteux M, Coulouarn C. Exosomal circRNAs: new players in the field of cholangiocarcinoma. Clin Sci (Lond). 2019;133(21):2239–2244. doi: 10.1042/CS20190940 31654054

[130] Pakarinen S, Varpe P, Carpelan A, et al. Mobile-CEA – a novel surveillance method for patients with colorectal cancer. Cancer Control. 2022;29:10732748221102780. doi: 10.1177/10732748221102780 35695276 PMC9209784

[131] Scarà S, Bottoni P, Scatena R. CA 19–9: biochemical and clinical aspects. Adv Exp Med Biol. 2015;867:247–260. 26530370 10.1007/978-94-017-7215-0_15

[132] Tang W, Fu K, Sun H, et al. CircRNA microarray profiling identifies a novel circulating biomarker for detection of gastric cancer. Mol Cancer. 2018;17(1):137. doi: 10.1186/s12943-018-0888-8 30236115 PMC6147053

[133] Hashemi M, Daneii P, Zandieh MA, et al. Non-coding RNA-mediated N6-methyladenosine (m(6)A) deposition: a pivotal regulator of cancer, impacting key signaling pathways in carcinogenesis and therapy response. Noncoding RNA Res. 2024;9(1):84–104. doi: 10.1016/j.ncrna.2023.11.005 38075202 PMC10700483

[134] Jarallah SJ, Aldossary AM, Tawfik EA, et al. GL67 lipid-based liposomal formulation for efficient siRNA delivery into human lung cancer cells. Saudi Pharm J. 2023;31(7):1139–1148. doi: 10.1016/j.jsps.2023.05.017 37273265 PMC10236467

[135] He AT, Liu J, Li F, et al. Targeting circular RNAs as a therapeutic approach: current strategies and challenges. Sig Transduct Target Ther. 2021;6(1):185. doi: 10.1038/s41392-021-00569-5 PMC8137869 34016945

[136] Huang HC, Lin CJ, Sheng YJ, et al. Instability of membranes containing ionizable cationic lipids: effects of the repulsive range of headgroups and tail structures. Colloids Surf B Biointerfaces. 2024;236:113807. doi: 10.1016/j.colsurfb.2024.113807 38417348

[137] Escudé Martinez de Castilla P, Estapé Senti M, Erkens S, et al. Reticuloendothelial system blockade does not enhance siRNA-LNP circulation or tumor accumulation in mice. Int J Pharm X. 2025;9:100324. doi: 10.1016/j.ijpx.2025.100324 40115963 PMC11925117

[138] Judge AD, Robbins M, Tavakoli I, et al. Confirming the RNAi-mediated mechanism of action of siRNA-based cancer therapeutics in mice. J Clin Invest. 2009;119(3):661–673. doi: 10.1172/JCI37515 19229107 PMC2648695

[139] Liu W, Zuo B, Liu W, et al. Long non-coding RNAs in non-small cell lung cancer: implications for preventing therapeutic resistance. Biochim Biophys Acta Rev Cancer. 2023;1878(6):188982. doi: 10.1016/j.bbcan.2023.188982 37734560

[140] Lu YY, Li Y, Chen ZL, et al. Genetic switch selectively kills hepatocellular carcinoma cell based on microRNA and tissue-specific promoter. Mol Cell Probes. 2024;77:101981. doi: 10.1016/j.mcp.2024.101981 39197503

[141] Kameda S, Ohno H, Saito H. Synthetic circular RNA switches and circuits that control protein expression in mammalian cells. Nucleic Acids Res. 2023;51(4):e24. doi: 10.1093/nar/gkac1252 36642090 PMC9976894

[142] Ning H, Liu G, Li L, et al. Rational design of microrna-responsive switch for programmable translational control in mammalian cells. Nat Commun. 2023;14(1):7193. doi: 10.1038/s41467-023-43065-w 37938567 PMC10632459

[143] Bray F, Laversanne M, Sung H, et al. Global cancer statistics 2022: GLOBOCAN estimates of incidence and mortality worldwide for 36 cancers in 185 countries. CA Cancer J Clin. 2024;74(3):229–263. doi: 10.3322/caac.21834 38572751

[144] Han B, Zheng R, Zeng H, et al. Cancer incidence and mortality in China, 2022. J Natl Cancer Cent. 2024;4(1):47–53. doi: 10.1016/j.jncc.2024.01.006 39036382 PMC11256708

[145] Perez M, Abisaad JA, Rojas KD, et al. Skin cancer: primary, secondary, and tertiary prevention. Part I. J Am Acad Dermatol. 2022;87(2):255–268. doi: 10.1016/j.jaad.2021.12.066 35176397

[146] Conn VM, Chinnaiyan AM, Conn SJ. Circular RNA in cancer. Nat Rev Cancer. 2024;24(9):597–613. doi: 10.1038/s41568-024-00721-7 39075222

[147] Wang S, Wang Z, Su H, et al. Effects of long-term culture on the biological characteristics and RNA profiles of human bone-marrow-derived mesenchymal stem cells. Mol Ther Nucleic Acids. 2021;26:557–574. doi: 10.1016/j.omtn.2021.08.013 34631285 PMC8479280

[148] Misir S, Wu N, Yang BB. Specific expression and functions of circular RNAs. Cell Death Differ. 2022;29(3):481–491. doi: 10.1038/s41418-022-00948-7 35169296 PMC8901656

[149] Li X, Yang L, Chen L-L. The biogenesis, functions, and challenges of circular RNAs. Mol Cell. 2018;71(3):428–442. doi: 10.1016/j.molcel.2018.06.034 30057200

[150] Wang Y, Zhang J, Yang Y, et al. Circular RNAs in human diseases. MedComm. 2024;5(9):e699. doi: 10.1002/mco2.699 39239069 PMC11374765

[151] Lin H, Conn VM, Conn SJ. Past, present, and future strategies for detecting and quantifying circular RNA variants. FEBS J. 2025;292(16):4073–4085. doi: 10.1111/febs.70012 39934961 PMC12366282

[152] Ma XK, Zhai SN, Yang L. Approaches and challenges in genome-wide circular RNA identification and quantification. Trends Genet. 2023;39(12):897–907. doi: 10.1016/j.tig.2023.09.006 37839990

[153] Vromman M, Anckaert J, Bortoluzzi S, et al. Large-scale benchmarking of circRNA detection tools reveals large differences in sensitivity but not in precision. Nat Methods. 2023;20(8):1159–1169. doi: 10.1038/s41592-023-01944-6 37443337 PMC10870000

[154] Verduci L, Tarcitano E, Strano S, et al. CircRNAs: role in human diseases and potential use as biomarkers. Cell Death Dis. 2021;12(5):468. doi: 10.1038/s41419-021-03743-3 33976116 PMC8113373

[155] Nielsen AF, Bindereif A, Bozzoni I, et al. Best practice standards for circular RNA research. Nat Methods. 2022;19(10):1208–1220. doi: 10.1038/s41592-022-01487-2 35618955 PMC9759028

[156] Zhang J, Hou L, Zuo Z, et al. Comprehensive profiling of circular RNAs with nanopore sequencing and CIRI-long. Nat Biotechnol. 2021;39(7):836–845. doi: 10.1038/s41587-021-00842-6 33707777

[157] Thorel L, Perréard M, Florent R, et al. Patient-derived tumor organoids: a new avenue for preclinical research and precision medicine in oncology. Exp Mol Med. 2024;56(7):1531–1551. doi: 10.1038/s12276-024-01272-5 38945959 PMC11297165

[158] Zenhausern R, Jang B, Schrader Echeverri E, et al. Lipid nanoparticle screening in nonhuman primates with minimal loss of life. Nat Biotechnol. 2025. doi: 10.1038/s41587-025-02711-y PMC13180654 40571690

[159] Apostolopoulos A, Kawamoto N, Chow SYA, et al. dCas13-mediated translational repression for accurate gene silencing in mammalian cells. Nat Commun. 2024;15(1):2205. doi: 10.1038/s41467-024-46412-7 38467613 PMC10928199

[160] Zhang J, Zhao F. Circular RNA discovery with emerging sequencing and deep learning technologies. Nat Genet. 2025;57(5):1089–1102. doi: 10.1038/s41588-025-02157-7 40247051

[161] Mostafa AM, Hamdy NM, Abdel-Rahman SZ, et al. Effect of vildagliptin and pravastatin combination on cholesterol efflux in adipocytes. IUBMB Life. 2016;68(7):535–543. doi: 10.1002/iub.1510 27251372

